# Sustainable Approaches to the Synthesis of Metallophthalocyanines in Solution

**DOI:** 10.3390/molecules26061760

**Published:** 2021-03-21

**Authors:** Gloria Zanotti, Patrizia Imperatori, Anna Maria Paoletti, Giovanna Pennesi

**Affiliations:** Istituto di Struttura della Materia (ISM) Consiglio Nazionale delle Ricerche (CNR), Via Salaria km 29.300, 00015 Monterotondo, Italy; patrizia.imperatori@ism.cnr.it (P.I.); annamaria.paoletti@ism.cnr.it (A.M.P.); gianna.pennesi@ism.cnr.it (G.P.)

**Keywords:** sustainability, alternative solvents, metallomacrocycles

## Abstract

This work aims to investigate more sustainable reaction conditions for the synthesis of metallophthalocyanines. Anisole, glycerol and their mixtures have been investigated as reaction media for the tetramerization of phthalonitriles. Acetates of three divalent first-transition metal cations, Co(II), Cu(II) and Zn(II), were used and several bases were tested, depending on the chosen substrates and reaction conditions, with a view to making the whole process more sustainable while ensuring its scalability. Unsubstituted phthalocyanines were synthesized to analyze the behavior of the different metal ions in terms of reactivity in the new reaction media, resulting in a general Cu > Co > Zn trend, while the nonpolar tetra-*tert*-butyl substitution was investigated to evaluate the synthesis of soluble derivatives in the new conditions. Furthermore, the potassium hydroxide (KOH)-aided statistical synthesis of the unsymmetrical 9(10), 16(17), 23(24)-tri-*tert*-butyl-2-iodophthalocyaninato zinc(II), starting from 4-*tert*-butylphthalonitrile and 4-iodophthalonitrile in a glycerol/anisole mixture, proceeded with a satisfactory 26% yield. Our results provide insights into the investigation of new reaction environments and the understanding of their strengths and weaknesses, with a view to further increasing the sustainability of the synthesis of metallomacrocycles with high added value while lowering their production cost.

## 1. Introduction

Sustainability keeps gaining increasing importance in many technological fields, such as energy production, conversion, and storage on large- and small-scales, and the synthesis of high-added value chemicals for a variety of technologies. Applying the green chemistry principles to synthetic processes makes them more affordable both from a sustainable and, as a consequence, an economical point of view, thanks to a rational design of the synthesis aimed at reducing wastes, choosing reagents and reactants in order to retain the most of their constituting atoms in the final products, and selecting appropriate and low-impacting reaction media.

Since solvents usually represent a significant portion of the whole material involved in a chemical process, their replacement for “greener” alternatives is an effective way to improve the sustainability of the entire synthesis. In principle, solvent-free conditions would be the optimum choice; however, most of the synthetic applications consistently benefit from the use of a solvent or a solvent mixture for several reasons. Dissolving reagents in a liquid medium (i) minimizes inhomogeneities both in the temperature of the reaction and in the reagents ratios, (ii) promotes the selectivity of the reaction due to interactions between the solvent molecules and the reagents and inducing the activation of specific molecular positions among others, and (iii) enables an easier handling, processing and purification of the resulting crude mixture [1,2].

Phthalocyanines have been among the most important family of dyes and pigments since their discovery, around 100 years ago. The central cavity of their aromatic structure can host a wide variety of metal ions, and the macrocycle periphery can be rationally tailored to tune their chemical, optical, and electronic properties according to their final application, resulting in an ever-increasing range of structures that are widely implemented in a variety of sectors, such as catalysis [3,4], gas sensing [5], photodynamic therapy [6], organic electronics [7,8] and hybrid photovoltaics [9,10,11,12]. The synthesis of phthalocyanines can be performed in several ways: a cheap and scalable protocol, currently used to synthesize industrially relevant pigments such as the phthalocyanine-blue, involves the reaction of phthalic anhydride with urea as the nitrogen source and a catalytic amount of ammonium heptamolybdate. On the lab scale, substituted and unsubstituted phthalocyanines are usually synthesized starting from phthalonitriles, either with or without a templating salt depending on the desired coordination of their central cavity. From the perspective of green chemistry, the atom economy, namely, the amount of atoms of the reagents that are retained in the final products, of the “phthalonitrile route” is consistently higher than that of the “phthalic anhydride route”. Nevertheless, it still often requires an organic base, commonly 1,8-diazabicyclo(5.4.0)undec-7-ene (DBU), to catalyze the reagent tetramerization and reaction media such as alkyl alcohols (pentanol, hexanol), dimethylaminoethanol (DMAE) nitrobenzene, chlorobenzene, quinoline and α-chloronaphthalene [13,14,15,16,17,18,19,20,21], most of which are non-environmentally friendly. Given their extreme technological importance, many unconventional synthetic approaches have been studied and developed in parallel with their applications to increase their yields and sustainability while reducing their production cost, such as (i) microwave-assisted [22] and UV-assisted [23,24] processes, which are usually much faster and cleaner than their “standard” counterparts but requiring specific instrumentation and lacking scalability, and (ii) room-temperature reactions promoted by strong organic bases such as lithium diisopropylamide (LDA) [25] and pre-formed lithium alcoholate solutions [26]. With a view to increase the sustainability of the phthalocyanine synthesis on the lab scale, we systematically studied the effect of solvent and base replacement on the yield and cost of the macrocyclization reaction of phthalonitrile and 4-*tert*-butylphthalonitile templated by three first-transition metal ions: Co(II), Cu(II) and Zn(II). We focused our attention on two main solvents, anisole, glycerol and their mixtures, for several reasons. The former is a low-cost, non-toxic and biodegradable compound that ranks very well in recent solvent selection guides [27,28] and can be obtained from renewable sources such as lignin and guaiacol [29,30,31]. Its aromatic nature can in principle guarantee the good solubility of a variety of phthalonitrile precursors, and its boiling point is suitable for macrocyclizations in solution. The latter is available on a large scale from the vegetable oil industry, is non-toxic, and can dissolve inorganic salts as well as a variety of organic molecules that would be immiscible with water [32].

Furthermore, we tested potassium hydroxide (KOH) as a possible and cheaper alternative to the use of DBU given its lower cost and wide availability, bearing in mind the possibility of side-reactions on the nitrile groups due to the strong nucleofilicity of the ^-^OH ion. When testing our alternative protocols, hereinafter named A-DBU (anisole + DBU), A-KOH (anisole + KOH) and GA-KOH (glycerol/anisole + KOH), both unsubstituted and 2,9(10),16(17),23(24)-tetra-*tert*-butyl phthalocyanines formation showed an evident dependence on the nature of the central metal: copper gave the best results in terms of yield, with values up to 76% for the KOH-aided tetramerization of phthalonitrile in anisole, and both its KOH-based synthetic protocols were up to 73.43 EUR/kg cheaper than standard methods, as highlighted in the cost analysis reported in Section 2.3. On the contrary, the reactivity of zinc was very poor whenever the inorganic base replaced DBU. Interestingly, when extending the GA-KOH approach to the synthesis of a non-symmetrically substituted zinc phthalocyanine, specifically 9(10), 16(17), 23(24)-tri-*tert*-butyl-2-iodophthalocyaninato zinc(II), the reaction provided the expected mixture of mono- and poly-iodinated phthalocyanines along with 2,9(10),16(17),23(24)-tetra-*tert*-butylphthalocyaninato zinc(II), obtained in higher yields than when reacting 4-*tert*-butylphthalointrile in the same conditions.

Our study, far from being exhaustive, aims to be a first step towards the investigation of alternative reaction protocols for the synthesis of state-of-the-art and novel phthalocyanines, with the aim of providing alternatives with increased sustainability and lower costs while preserving their effectiveness.

## 2. Results and Discussion

### 2.1. Synthesis

#### 2.1.1. Unsubstituted and 2,9(10),16(17),23(24)-tetrasubstituted Phthalocyanines

**Scheme 1:**
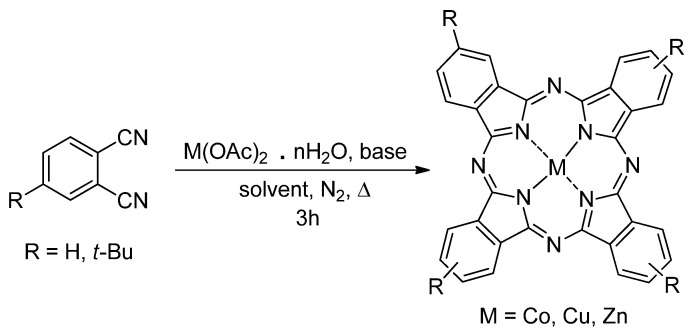
General synthetic procedure for unsubstituted and tetra-*tert*-butyl phthalocyanines. n = 1 (Cu), 2 (Zn), 4 (Co). General conditions: 1 eq phthalonitrile, 0.30 eq hydrated salt. Δ = 135 °C (DMAE), 154 °C (anisole), 175 °C (anisole/glyerol).

The general reaction for the synthesis of unsubstituted and tetrasubstituted derivatives is sketched in Scheme 1.

Anisole has been used with DBU to decouple any solvent effect with changing the base, and with KOH in order to move towards greener and cheaper conditions. When using pure glycerol at temperatures above 130 °C, a consistent amount of phthalonitrile crystallized on the neck of the reaction flask. The addition of anisole as a cosolvent, whose solubility in glycerol is tabulated as a function of quantity and temperature [33], favored the dissolving of the organic reagent, and a 10% *w/w* solution proved to be suitable to our scopes. In parallel with the alternative protocols reactions in standard conditions, i.e., in dimethylaminoethanol with DBU as the base, have been carried out for comparison for all the derivatives. In all cases, the yields have been calculated after washing the crudes with diluted HCl, water and methanol, and purifying the resulting solids in a Soxhlet extractor with acetic acid. To reduce the number of variables we always worked with acetate salts.

We firstly focused on the synthesis of unsubstituted cobalt(II), copper(II) and zinc(II) phthalocyanines, to evaluate the influence of the central metal on our new reaction conditions. The best yielding ones are summarized in Table 1.

The nature of our products has been confirmed by standard spectroscopic techniques and powder X-ray diffraction. The latter will be discussed in Section 2.2; the UV-Vis spectra in tetrahydrofuran (THF) showed phthalocyanine’s typical absorption Q and Soret bands, and are reported in the Supplementary Materials as Figure S10a,b. The narrow shape of the Q bands indicates the absence of aggregation in solution, in accordance with the coordinating nature of THF. Furthermore, the structure of ZnPc was confirmed by ^1^H NMR (Figure S1), in which the two multiplets centered at 9.44 and 8.28 ppm can be assigned to the protons of the aromatic ring. As a first comment, all the tested alternative conditions depend on the templating metal much more than the standard protocols. As expected, given its 3d^10^ shell, Zn(II) is in general the less reactive ion, and did not react at all in KOH-based procedures, while in the same conditions copper gave the best results. The generally lower yield of the DBU-assisted tetramerization in anisole with respect to that in DMAE, even if performed at slightly higher temperatures, might be due to the weak polar aprotic nature of the solvent that could not favor an efficient solubilization of acetate salts nor a suitable environment for the coordination of the +2 metal ion to the reaction intermediates. Contrary to what was expected, the yield for the formation of CuPc in the A-DBU conditions was the lowest, and the color of the resulting mixture was dark-brown rather than purple as in other cases. It is probable that the chemical properties of the solvent allow the Cu(II) ions to catalyze some oxidation processes rather than templating the formation of the macrocycle. The better results obtained with cobalt and zinc in the same conditions may support this hypothesis. In the A-KOH conditions, CoPc was formed only in a 24% yield, less than when we worked with the glycerol/anisole mixture, due to the very scarce solubility of the inorganic base in anisole, along with the probable reduced complexation capability of the cobalt cation. The formation of CuPc in the same conditions provided more satisfactory results, consistently with the templating properties of Cu(II), which is known to provide the highest yields for the synthesis of phthalocyanines. The slightly lower yield obtained with the GA-KOH approach with respect to the A-KOH approach is mainly due to the side-reactions of phthalonitrile that will be discussed later in this paragraph.

After evaluating the formation of unsubstituted phthalocyanines, we extended our approach to the synthesis of tetra-*tert*-butyl-substituted derivatives, following the same procedures described above. A summary of the best-yielding reactions is reported in Table 2.

Generally speaking, the yields and yield ratios were lower than those of the unsubstituted derivatives, in spite of the better solubility of 4-*tert*-butylphthalonitrile in the reaction media. Contrary to the CoPc general trend, *t*-Bu_4_CoPc was synthesized in appreciable yields only with the A-DBU protocol. Instead, *t*-Bu_4_CuPc was successfully synthesized using the A-KOH and GA-KOH approaches, even though in lower yields with respect to the standard procedure, as a further confirmation that Cu(II) is the most reactive among the chosen metal ions. The A-KOH protocol was found to benefit from adding a small amount of ethanol (1 drop per 1.63 mmol of phthalonitrile), which promoted a better availability of KOH in the reaction medium along with the partial formation of the ethanoate anion, and raised the yield from 10 to 43%. On the contrary, adding an excess of ethanol lowered the yield to 32%, as it favored the undesired formation of hydration byproducts. *t*-Bu_4_ZnPc synthesized with the GA-KOH procedure was obtained as statistical mixtures of regioisomers, clearly visible by the multiplicity of the methyl signal centered at 1.76 ppm in the ^1^H NMR spectrum of a diluted sample in DMSO-d_6_ (Figure S2). By extension, the formation of regioisomeric mixtures for the Co(II) and Cu(II) tetrasubstituted derivatives is very probable, although not experimentally proven. As for the unsubstituted phthalocyanines, the UV-Vis spectra of THF solutions were compatible with the structure of our target products, and are reported in Figure S11.

**Scheme 2:**
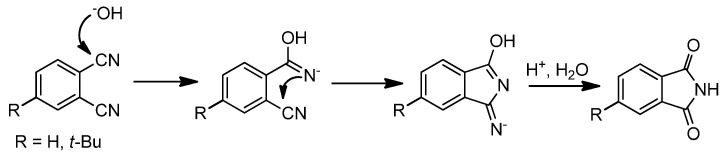
Proposed hydration reaction of phthalonitriles to phthalimides.

For both unsubstituted and tetrasubstituted phthalocyanines synthesized in the GA-KOH conditions, the concomitant formation of (4-*tert*-butyl)phthalimide could not be avoided and was confirmed by ^1^H-NMR (see Figure S4, S5). The hydration of nitrile groups in polar environments in the presence of ^-^OH ions is reported in the literature [34,35], and proceeds through a nucleophilic attack of the hydroxide anion on the nitrile carbon group. As sketched in Scheme 2, in the case of *o*-dicyanobenzenes, a five-membered ring closure can be promoted by an intramolecular nucleophilic attack followed by acidic hydrolisis, as reported in the presence of several nucleophiles such as ethanoates [36,37].

#### 2.1.2. Statistical Synthesis of Unsymmetrical Phthlaocyanines

**Scheme 3:**
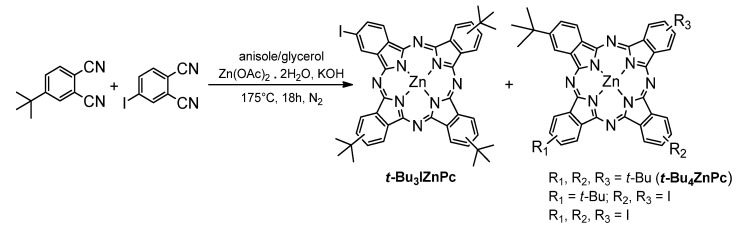
Statistical reaction between 4-*tert*-butylphthalonitrile and 4-iodophthalonitrile.

Lastly, we extended our approach to the synthesis of unsymmetrical phthalocyanines, in which more than one type of phthalonitrile is involved and a variety of products with different degrees of substitutions is expected, to evaluate any variations in terms of yield and imbalance towards one of the possible products. Specifically, we investigated the statistical synthesis of tri-*tert*-butyl-iodophthalocyaninatozinc(II) (**t-Bu_3_IZnPc**), starting from 4-*tert*-butylphthalonitrile and 4-iodophthalonitrile, given its versatility as an intermediate for the synthesis of a variety of compounds for several technological applications [38,39,40]. The related reaction is reported in Scheme 3; the glycerol/anisole mixture was chosen as solvent, with KOH as a base and a 5:1 ratio between 4-*tert*-butylphthalonitrile and 4-iodophthalonitrile, respectively.

Surprisingly, differently from the tetramerization of 4-*tert*-butylphthalonitrile in the same conditions, the reaction started quickly and the presence of the expected product mixture, including **t-Bu_4_ZnPc**, was confirmed by thin-layer chromatography (TLC). After purification by column chromatography on silica gel, we isolated **t-Bu_3_IZnPc** in a satisfactory 26% yield, along with a 41% yield for the tetra-*tert*-butyl derivative, similarly to what we obtained in our laboratory using standard procedures with the same phthalonitriles molar ratio. ^1^H NMR and electrospray ionization mass spectrometry (ESI-MS) confirmed the structure of both compounds. Although we cannot provide a rigorous explanation for this evidence, regarding the unsymmetrical derivatives we may infer that 4-iodophthalonitrile, which is slightly more reactive than an alkyl-phthalonitrile, may efficiently undergo nucleophilic attack on one nitrile group, and form a sufficiently reactive intermediate to attack 4-*tert*-butylphthalonitrile and involve it in the tetramerization process.

### 2.2. XRD Characterization

**Figure 1:**
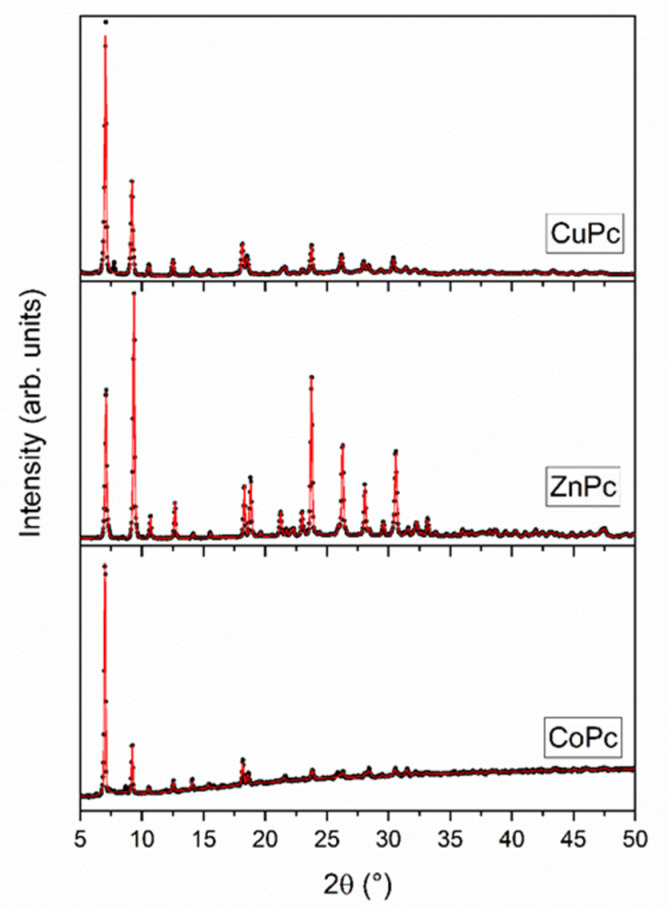
X-ray diffraction (XRD) patterns of Cu-, Zn- and Co-phthalocyanine. The red line is the Rietveld fit of the experimental data.

X-ray diffraction (XRD) spectra are a useful further confirmation of the structure of our products. The powder XRD patterns of Cu-, Zn- and Co-phthalocyanine are reported in Figure 1. The three compounds were identified as β phase, according to the JCPDS cards n. 39-1881, 39-1882 and 14-0948 for the βCu-, βZn- and βCo-phthalocyanine, respectively. The Rietveld fit of the data, performed using the monoclinic structure of the β phase, is shown in Figure 1 as well (red line). The lattice parameters obtained from the fit are reported in Table 3.

**Figure 2:**
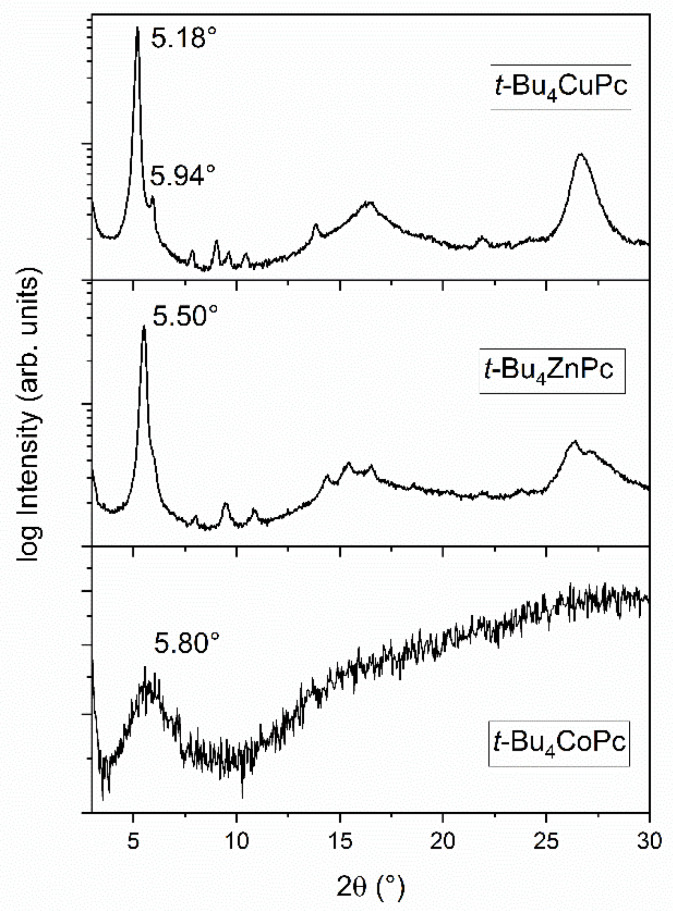
XRD patterns of tetra-*tert*-butyl-substituted Cu-, Zn- and Co-phthalocyanine.

The XRD patterns of tetra-*tert*-butyl-substituted Cu-, Zn- and Co-phthalocyanine are shown in Figure 2. The substituted phthalocyanines present a lack of reflections when compared to the corresponding non substituted ones, indicating a lower crystallinity, even though the logarithmic scale of intensity reveals the presence of weak reflections. Moreover, data are probably an average of the four *t*-Bu_4_MPc isomers.

An intense low-angle reflection at 2θ of 5.50° (d = 17.04 Å) and 5.18° (d = 16.07 Å) for *t*-Bu_4_ZnPc and *t*-Bu_4_CuPc, respectively, is present. The *t*-Bu_4_CoPc sample only shows a weak broad peak at 2θ = 5.80° (d ~ 15.24 Å), indicating an amorphous structure.

The crystal structures of *t*-Bu_4_MPc are not reported in the literature and the few data on powder samples are often discordant. Lebedeva et al. [41] presented powder XRD data on either α- and β- *t*-Bu_4_ZnPc phases, indicating for both phases a d_100_ spacing of 16.97Å, in quite good agreement with our value. Dong et al. [42] showed an XRD pattern for powder ***t*-**Bu_4_ZnPc with only a low angle peak at 2θ = 6.0°, which was a slightly higher value than ours.

G. Sfyri et al. [43] reported the grazing incidence XRD pattern of *t*-Bu_4_CuPc film, showing two low-angle reflections at 5.18° and 5.92°, well in agreement with our data (see Figure 2).

### 2.3. Cost Analysis

Since we have stressed the importance of lowering the fabrication costs of our target materials while increasing their sustainability, we propose a cost-per-kilogram analysis for our new experimental conditions and compare the obtained results with the “standard” protocol that we used and with the current price of CoPc, CuPc, ZnPc. The comparison among the tetra-*tert*-butylated phthalocyanines is not reported because the high price/kg of 4-*tert*-butylphthalonitrile, along with generally lower yields with respect to the standard protocol, far outweighs the economic advantage of using less expensive chemicals. The total material cost (in EUR) for our phthalocyanines has been estimated according to a paper published in 2013 by Osedach et al. [44]. For the sake of simplicity, Merck has been chosen as the only supplier for reagents, solvents and purification materials. In all cases, the bulkiest batches of suitable purity available on the online catalogs have been considered, the list of which is reported in Table S1 in the supporting information, along with further technical details. Moving from Lab to Fab requires a complete cost analysis that must include parameters like energy, facilities maintenance and personnel costs, taxes, and other charges that we have not taken into account. Furthermore, we are aware that large-scale synthesis may significantly differ from small-scale preparations in many ways (homogeneity of the reaction mixture, temperature uniformity, type of stirring, purification methods), but as a first approximation, the costs of our syntheses have been estimated by assuming linearity when scaling the procedure from grams to kilograms. Prices were estimated by calculating the required amounts of chemicals to produce and purify 1 kg of the desired product in the obtained yield, and multiplying them by the respective prices. The Soxhlet purification has not been included in the discussion since the removal of impurities can be carried out with the same solvent batch multiple times. The resulting costs include reagents, solvents and purification materials, and are reported in Table 4, given as EUR per kilogram of synthesized product.

As a general remark, the reagents and purification costs increase whenever the yield lowers with respect to the standard conditions, given the increased amount of chemicals needed to obtain 1 kg of product. Reagents for the synthesis of CoPc in standard conditions have a higher cost than those of CuPc and ZnPc due to the higher price of cobalt acetate. Conversely, in some cases the alternative solvents positively affect the overall price since their cost per kg is considerably lower than that of DMAE. It is noteworthy to point out the huge impact that the work-up and purification steps have on the price of the final product, as highlighted by the differences in the related net and gross total costs. The optimization of any synthetic process is mandatory in order to scale it up, and materials recycling is an essential part of it, so we are confident that most of the purification solvents, e.g., methanol, could be recovered and used more than once, significantly lowering the overall cost and waste production. Among the others, both syntheses of CuPc with KOH are less costly than their standard counterpart, by up to 73.43 EUR/kg, and are much cheaper than the alternatives on the market even when the purification costs are included in the price. Furthermore, the similarity between the net prices of ZnPc is encouraging and suggests that a careful optimization of the reaction parameters, along with a rational strategy to minimize the impact of the workup in terms of costs, could further lower the economic impact of the A-DBU protocol.

## 3. Materials and Methods

All reagents and solvents were purchased from Merck Life Science S.r.l (Milano, Italy), TCI Chemicals (Zwijndrecht, Belgium) and Carlo Erba Reagents (Cornaredo, Italy) and used without further purification. Reactions were purged and refilled three times, performed under nitrogen and monitored by thin-layer chromatography (TLC) employing a polyester layer coated with 250 mm F254 silica gel. Chromatographic purifications, when needed, were performed using silica gel 60A 35–70 μ and several mobile phases, depending on the nature of the target molecule. ^1^H spectra were recorded on a Bruker AVANCE 600 NMR spectrometer (Billerica, MA, USA) operating at a proton frequency of 600.13 MHz in DMSO-*d_6_*; chemical shifts (δ) are given in ppm relative to TMS. UV-Vis spectra were recorded on a Perkin-Elmer Lambda 950 UV-vis/NIR spectrophotometer (Perkin Elmer Italia, Milano, Italy) using THF as solvent. Infrared spectra were recorded on a Shimadzu FT-IR prestige-21 spectrometer (Kyoto, Japan) using an attenuated total reflectance (ATR) unit. ESI-MS -spectra were recorded at the Toscana Life Science facility with a Thermo Fisher Scientific Q-Exactive Plus (Geel, Belgium). The samples have been analyzed by direct infusion in positive mode with a spray voltage of 3.5 kV, sheat gas 10 a.u., and a flow rate of 5 ul/min. XRD measurements were carried out on a Seifert XRD 3003 TT diffractometer (Ahrensburg, Germany) in the Bragg–Brentano geometry, using Cu Kα radiation (λ = 1.5418 Å). θ-2θ scans were performed in the 2θ range 5–50° with step size 0.04° and counting 8 s/step. Rietveld refinements were done using GSAS II software [45].

### 3.1. General Synthesis of Unsubstituted Phthalocyanines

In a two-necked 50 mL round bottom flask equipped with a reflux condenser and a magnetic stirrer, 1.0 g (1 eq) of phthalonitrile, 0.30 eq of hydrated salt and two Pasteur-drops (approx. 32 mg, 0.21 mmol, 0.027 eq) of DBU (or 0.21 eq of KOH) were added and stirred in 5 mL of solvent (or in a 5.5 g of anisole/glycerol mixture) under reflux for 3 h. The mixture was then cooled, treated with 15 mL of HCl 1N, filtered and washed with water (2 × 20 mL) and methanol (2 × 15 mL). It was then purified in a Soxhlet extractor with glacial acetic acid until the extracts were colorless.

CoPc: purple microcrystalline powder; UV (THF) *λ*_abs_ 324, 593, 656 nm.

CuPc: purple microcrystalline powder; UV (THF) *λ*_abs_ 342, 601, 666 nm. *scarcely soluble

ZnPc: purple microcrystalline powder; UV (THF) *λ*_abs_ 342, 601, 666 nm [46]; ^1^H NMR (DMSO-d_6_, 600 MHz) δ 9.44 (8H, m), 8.28 (8H, m).

### 3.2. General Synthesis of 2,9(10),16(17),23(24)-Tetra-tert-butyl-Substituted Phthalocyanines

In a two-necked 50 mL round bottom flask equipped with a reflux condenser and a magnetic stirrer, 1.0 g (5.43 mmol, 1 eq) of 4-*tert*-butylphthalonitrile, 0.30 mmol of hydrated salt and two Pasteur-drops (approx 32 mg, 0.21 mmol, 0.0387 eq) of DBU (or 0.21 to 0.3 eq of KOH) were added and stirred in 5 mL of solvent (or in a 5.5 g of anisole/glycerol mixture) under reflux for 3 h. The mixture was then cooled, treated with 15 mL of HCl 1N, filtered, and washed with water (2 × 20 mL) and methanol (2 × 15 mL). The resulting Zn and Co crudes were then purified by filtration on a silica pad (10g) using DCM as a solvent, while the Cu derivative was washed with hot methanol until washings were pale yellow/colorless.

*t*-Bu_4_CoPc: shiny purple crystals; UV (THF) λ_abs_ 328, 598, 661 nm; ESI *m/z* 795.84 [M]^+^

*t*-Bu_4_CuPc: shiny purple crystals; UV (THF) λ_abs_ 344, 605, 671 nm; ESI *m/z* 800.33 [M]^+^

*t*-Bu_4_ZnPc: shiny purple crystals; UV (THF) λ_abs_ 348, 606, 672 nm [47]; ^1^H NMR (DMSO-d_6_, 600 MHz) δ 9.38–9.16, (8H, m), 8.32–8.24 (4H, m), 1.76 (36H, m, C-(CH_3_)_3_), regioisomers); ESI *m/z* 802.83 [M]^+^

### 3.3. Synthesis of Tri-tert-butyl-iodophthalocyaninatozinc(II)

In a two-necked 50 mL round bottom flask equipped with a reflux condenser and a magnetic stirrer, 520 mg (5 eq, 2.822 mmol) of 4-*tert*-butylphthalonitrile, 140 mg of 4-iodophthtalonitrile (0.55 mmol, 1 eq), 120 mg (0.688 mmol, 1.25 eq) of Zn(OAc)_2_ and 50 mg KOH (0.89 mmol, 2.62 eq) were mixed in a 10% *w*/*w* anisole-glycerol mixture (5.5 g) at 175 °C for 18 h. After removing anisole under reduced pressure, the mixture was treated with HCl 1N and the resulting solid was washed with water and methanol to provide 380 mg of crude. The desired product and 2,9(10),16(17),23(24)-tetra-*tert*-butyl phthalocyanine were purified by column chromatography on silica gel using several petroleum ether–THF mixtures (98:2 to 4:1), and they were obtained in 26% (125 mg) and 41% (180 mg) yields, respectively.

*t*-Bu_3_IZnPc: shiny purple crystals; UV (THF) λ_abs_ 350, 606, 672 nm; ESI *m/z* 872.67 [M]^+^.

## 4. Conclusions

To summarize, we have explored the potentials of greener synthetic approaches for the Co(II), Cu(II) and Zn(II) templated synthesis of unsubstituted and 2,9(10),16(17),23(24)-tetra-*tert*-butylphthalocyanines in solution, either using DBU or KOH as bases. Our experimental protocols showed a remarkable dependence on the nature of the metal ions, with the best performances obtained by Cu(II) consistently with its excellent templating properties. The yields obtained for the synthesis of CuPc with both KOH-assisted approaches, even though slightly lower than that in standard conditions, are economically advantageous, while the synthesis of ZnPc with the A-DBU protocol, a few EUR/kg more expensive than that in the DMAE net of the purification costs, could become so with a further optimization of the procedure. 2,9(10),16(17),23(24)-tetra-*tert*-butylphthalocyanines were obtained in appreciable yields only with the A-DBU protocol. Further optimizations of the KOH-aided approaches, as in the case of the A-KOH synthesis of *t*-Bu_4_CuPc, may provide better results. When extending the GA-KOH protocol to the synthesis of unsymmetrical *t*-Bu_3_IZnPc, the result exceeded our expectations and confirmed the potential of our approach, which will be further investigated to better understand the involved phenomena and allow its extension to the synthesis of other macrocyclic targets. To our knowledge, this is the first work in which the results of a systematic synthesis of metallophthalocyanines in solution with more environmentally sustainable reaction conditions are analyzed, and, although not exhaustive, in our opinion it shows considerable potential for application, in principle even on a large scale.

## Figures and Tables

**Table 1:** Best-yielding reactions for the considered unsubstituted phthalocyanines.

Product	DMAE-DBU	A-DBU	A-KOH	GA-KOH
CoPc	87%	72%	24%	37%
CuPc	83%	49%	76%	73% *
ZnPc	75%	56%	n/a **	n/a **

* With respect to the experimental procedure described in 3.1, the best result was achieved with 0.27 eq KOH. ** No formation of the expected product was observed.

**Table 2:** Best-yielding reactions for the considered tetra-*tert*-butylphthalocyanines.

Product	DMAE-DBU	A-DBU	A-KOH	GA-KOH
*t*-Bu_4_CoPc	62%	50%	traces	traces
*t*-Bu_4_CuPc	58%	38%	43% *	26%
*t*-Bu_4_ZnPc	49%	10%	n/a **	16%

Yields have been calculated after chromatographic purification for *t*-Bu_4_CoPc and *t*-Bu_4_ZnPc, and after washings with hot methanol for *t*-Bu_4_CuPc. * Addition of catalytic amount of 95% ethanol. ** No formation of the expected product was observed.

**Table 3:** Lattice parameters of Cu-, Zn- and Co-phthalocyanine.

	a (Å)	c (Å)	c (Å)	β (°)
CuPc	14.698 (1)	4.8137(8)	19.595(2)	121.06(1)
ZnPc	14.569(1)	4.8676(3)	19.279(2)	120.62(1)
CoPc	14.642(2)	4.804(2)	19.496(3)	120.60(1)

**Table 4:** Cost analysis for unsubstituted phthalocyanines.

Product	Protocol	Reagents (EUR/kg)	Solvents (EUR/kg)	Workup/Purification (EUR/kg)	Total (EUR/kg)	Total (no workup/purification) (EUR/kg)
CoPc	standard	238.57	221.08	356.03	815.68	459.65
	A-DBU	340.78	165.43	419.42	925.63	506.21
	A-KOH	1016.34	492.00	1247.47	2755.81	1508.34
	GA-KOH	671.47	240.17	835.75	1747.39	911.74
CuPc	standard	119.62	228.45	368.81	716.88	348.07
	A-DBU	199.41	241.56	612.47	1053.44	440.97
	A-KOH	131.02	157.82	400.15	688.99	288.84
	GA-KOH	134.11	100.53	408.81	643.45	234.64
ZnPc	standard	135.03	252.52	408.32	785.97	387.55
	A-DBU	179.19	212.43	538.64	930.26	391.62

## Data Availability

The data presented in this study are available in this article and in the Supplementary Materials file.

## Supplementary Materials

The following are available online. Figure S1: 1H NMR of ZnPc, Figure S2: 1H NMR of t-Bu4ZnPc, Figure S3: 1H NMR of t-Bu3IZnPc, Figure S4: 1H NMR of phthalimide, Figure S5: 1H NMR of 4-tert-butylphthalimide, Figure S6: ESI spectrum of t-Bu4CoPc, Figure S7: ESI spectrum of t-Bu4CuPc, Figure S8: ESI spectrum of t-Bu4ZnPc, Figure S9: ESI spectrum of t-Bu3IZnPc, Figure S10: UV-Vis spectra of unsubstituted phthalocyanines, Figure S11: UV-Vis spectrum of (t-bu)4substituted phthalocyanines, Figure S12: UV-Vis spectrum of t-bu3IZnPc, Figure S13: IR spectrum of CoPc, Figure S14: IR spectrum of CuPc, Figure S15: IR spectrum of ZnPc, Figure S16: IR spectrum of t-Bu4CoPc, Figure S17: IR spectrum of t-Bu4CuPc, Figure S18: IR spectrum of t-Bu4ZnPc, Figure S19: IR spectrum of t-bu3IZnPc, Figure S20: flowchart for the synthesis of 1 kg of CoPc in standard conditions, Figure S21: flowchart for the synthesis of 1 kg of CuPc in standard conditions, Figure S22: flowchart for the synthesis of 1 kg of ZnPc in standard conditions, Figure S23: flowchart for the synthesis of 1 kg of CoPc in A-DBU conditions, Figure S24: flowchart for the synthesis of 1 kg of CuPc in A-DBU conditions, Figure S25: flowchart for the synthesis of 1 kg of ZnPc in A-DBU conditions, Figure S26: flowchart for the synthesis of 1 kg of CoPc in A-KOH conditions, Figure S27: flowchart for the synthesis of 1 kg of CuPc in A-KOH conditions, Figure S28: flowchart for the synthesis of 1 kg of CoPc in GA-KOH conditions, Figure S29: flowchart for the synthesis of 1 kg of CuPc in GA-KOH conditions, Table S1: Overview of the quoted materials.

## Sample Availability

Samples of all the synthesized compounds are available from the authors.
