# Supplementary material for: Sustainable Approaches to the Synthesis of Metallophthalocyanines in Solution
Source: Molecules. 2021 Mar 21;26(6):1760. doi: 10.3390/molecules26061760 (PMC8003941; doi:10.3390/molecules26061760)

SUPPLEMENTARY MATERIALS

Sustainable approaches to the synthesis of metallophthalocyanines in solution

Gloria Zanotti, Patrizia Imperatori, Anna Maria Paoletti and Giovanna Pennesi

Index

Figure S1: ^{1}H NMR of ZnPc..p.3

Figure S2: ^{1}H NMR of *t*-Bu_4ZnPc..p.4

Figure S3: ^{1}H NMR of *t*-Bu_3IZnPc..p.5

Figure S4: ^{1}H NMR of phthalimide...p.6

Figure S5: ^{1}H NMR of 4-*tert*-butylphthalimide..p.7

Figure S6: ESI-MS spectrum of *t*-Bu_4CoPc...p.8

Figure S7: ESI-MS spectrum of *t*-Bu_4CuPc...p.9

Figure S8: ESI-MS spectrum of *t*-Bu_4ZnPc...p.10

Figure S9: ESI-MS spectrum of *t*-Bu_3IZnPc..p.11

Figure S10: UV-Vis spectra of unsubstituted phthalocyanines in THF...p.12

Figures S11-S12: UV-Vis spectra of (*t*-Bu)$_4$substituted phthalocyanines and (*t*-bu)$_3$IZnPc in THF................p.13

Figures S13-S14: IR spectra of CoPc and CuPc...p.14

Figures S15-S16: IR spectra of ZnPc and *t*-Bu_4CoPc..p.15

Figures S17-S18: IR spectra of *t*-Bu_4CuPc and *t*-Bu_4ZnPc..p.16

Figure S19: IR spectrum of *t*-Bu_3IZnPc..p.17

Cost analysis...pp.18-28

Table S1: Overview of the quoted materials...p.18

Figure S20: Flowchart for the synthesis of 1 kg of CoPc in standard conditions..p.19

Figure S21: Flowchart for the synthesis of 1 kg of CuPc in standard conditions..p.20

Figure S22: Flowchart for the synthesis of 1 kg of ZnPc in standard conditions..p.21

Figure S23: Flowchart for the synthesis of 1 kg of CoPc in A-DBU conditions..p.22

Figure S24: Flowchart for the synthesis of 1 kg of CuPc in A-DBU conditions..p.23

Figure S25: Flowchart for the synthesis of 1 kg of ZnPc in A-DBU conditions..p.24

Figure S26: Flowchart for the synthesis of 1 kg of CoPc in A-KOH conditions..p.25

Figure S27: Flowchart for the synthesis of 1 kg of CuPc in A-KOH conditions..p.26

Figure S28: Flowchart for the synthesis of 1 kg of CoPc in GA-KOH conditions...p.27

Figure S29: Flowchart for the synthesis of 1 kg of CuPc in GA-KOH conditions...p.28

Figure S1: ZnPc ^{1}H (DMSO-*d6*).

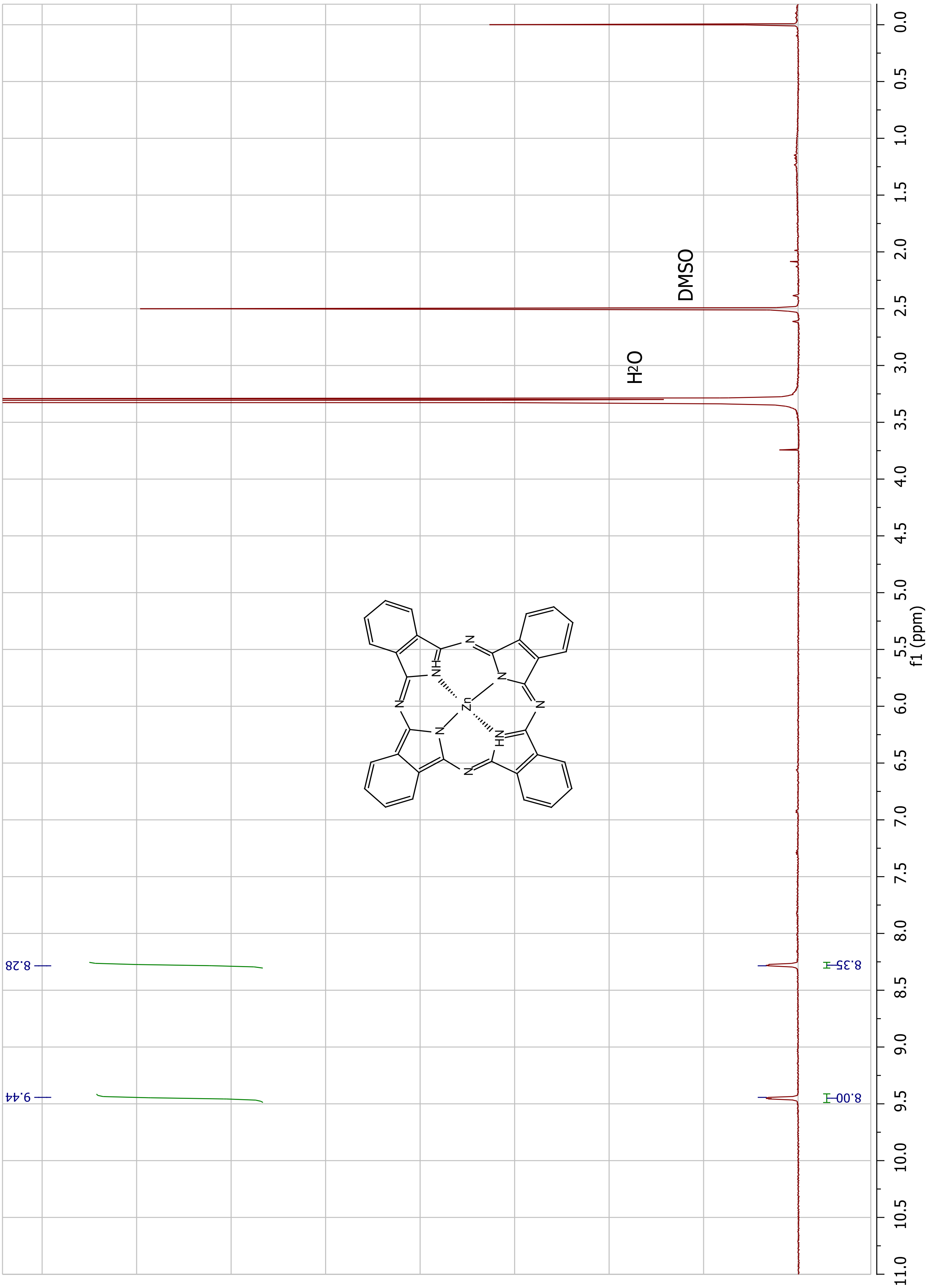

Figure S2: t-Bu$_4$ZnPc ^{1}H (DMSO-*d6*).

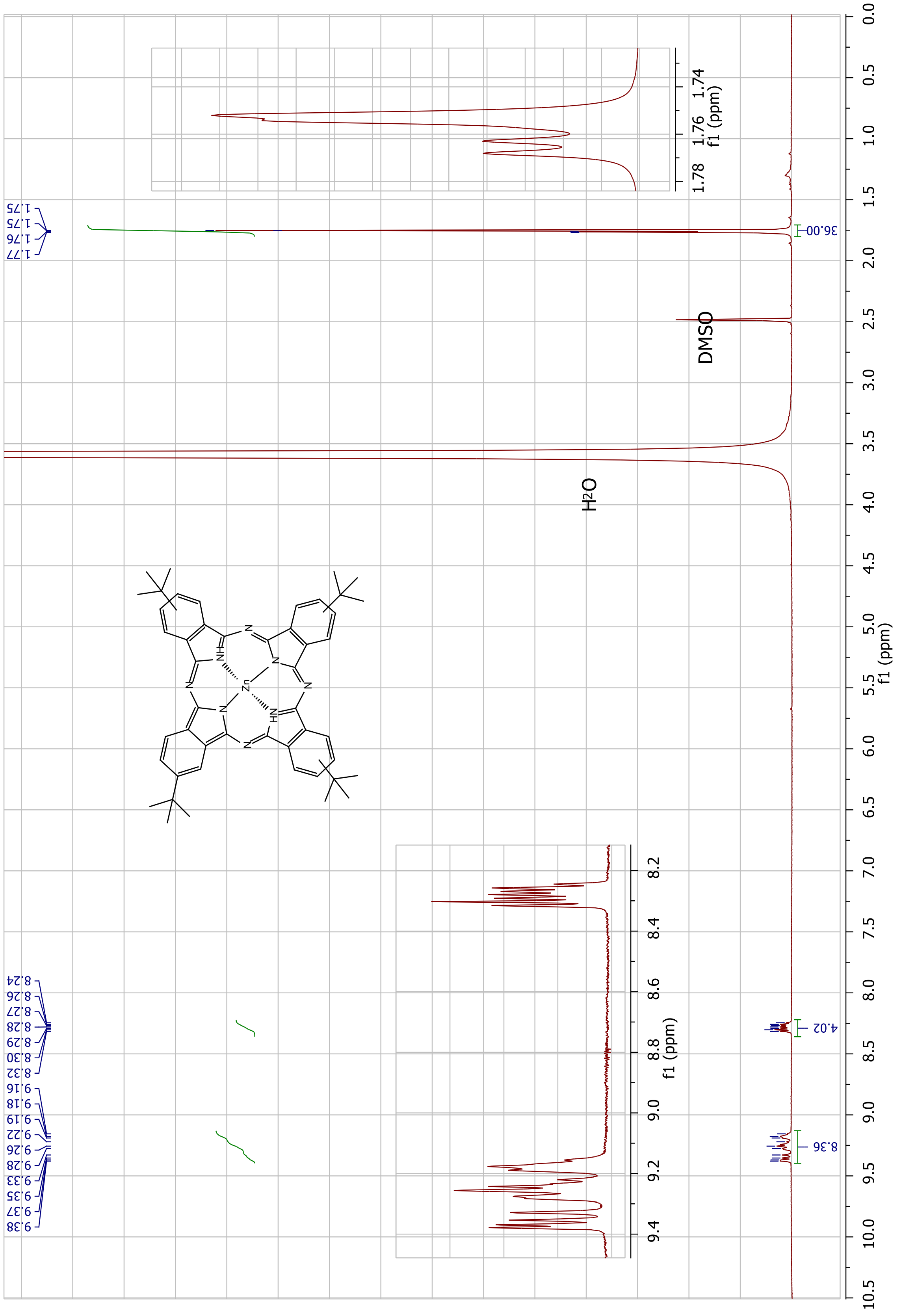

Figure S3: *t*-Bu_3IZnPc ^{1}H (DMSO-*d6*).

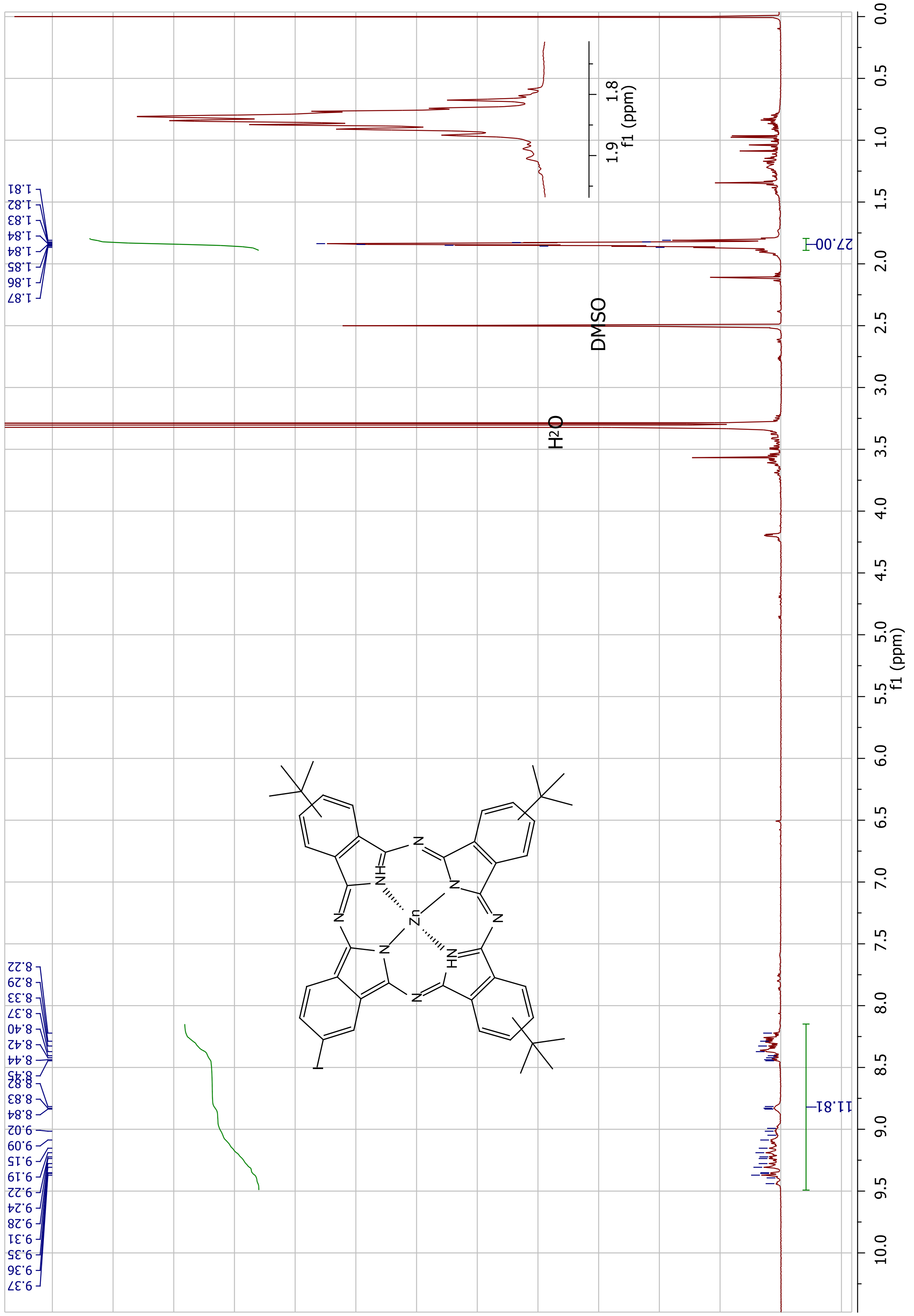

Figure S4: Phthalimide ^{1}H NMR (DMSO-*d6*).

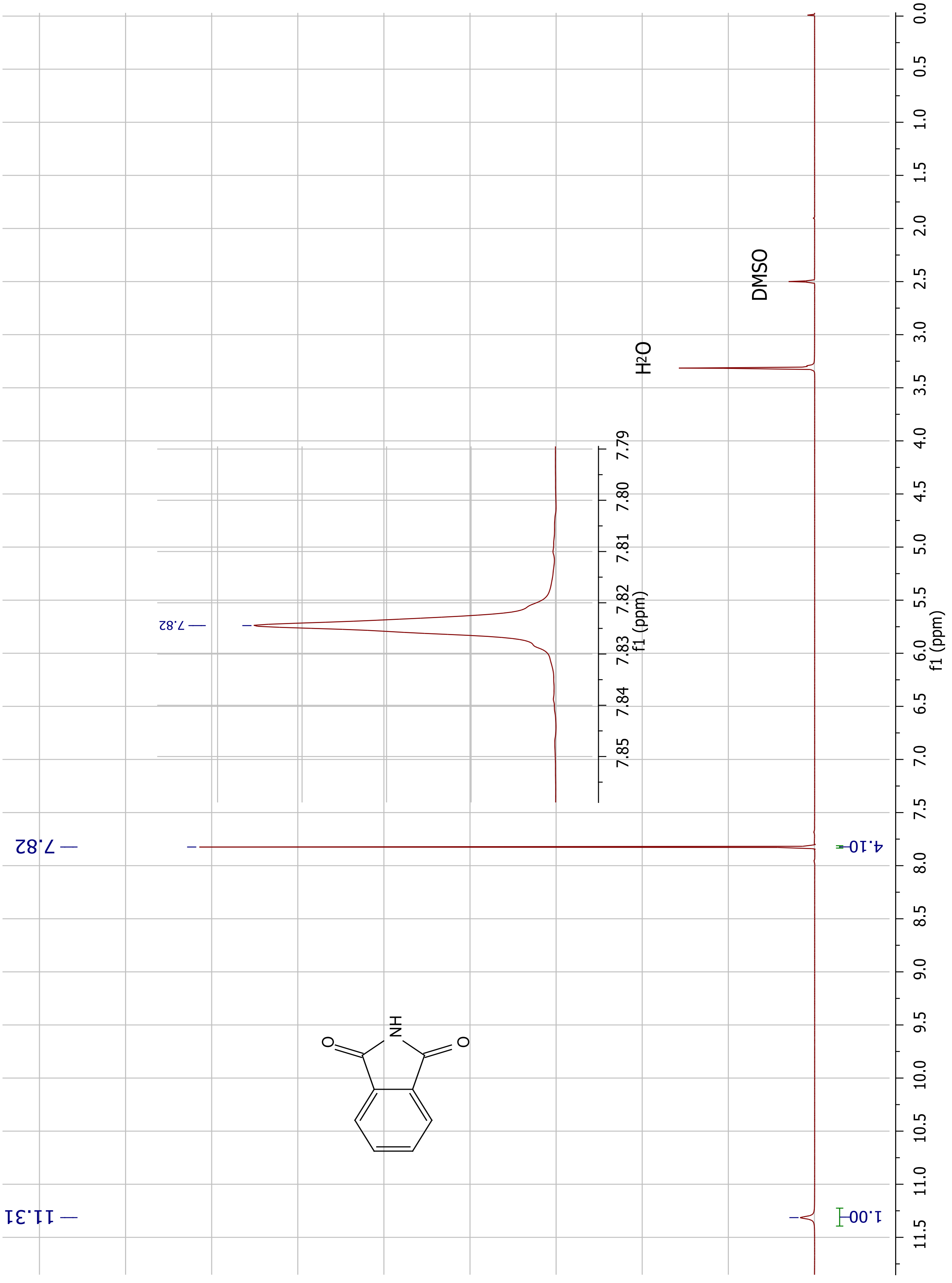

Figure S5- 4-*tert*-butylphthalimide ^{1}H NMR (DMSO-*d6*).

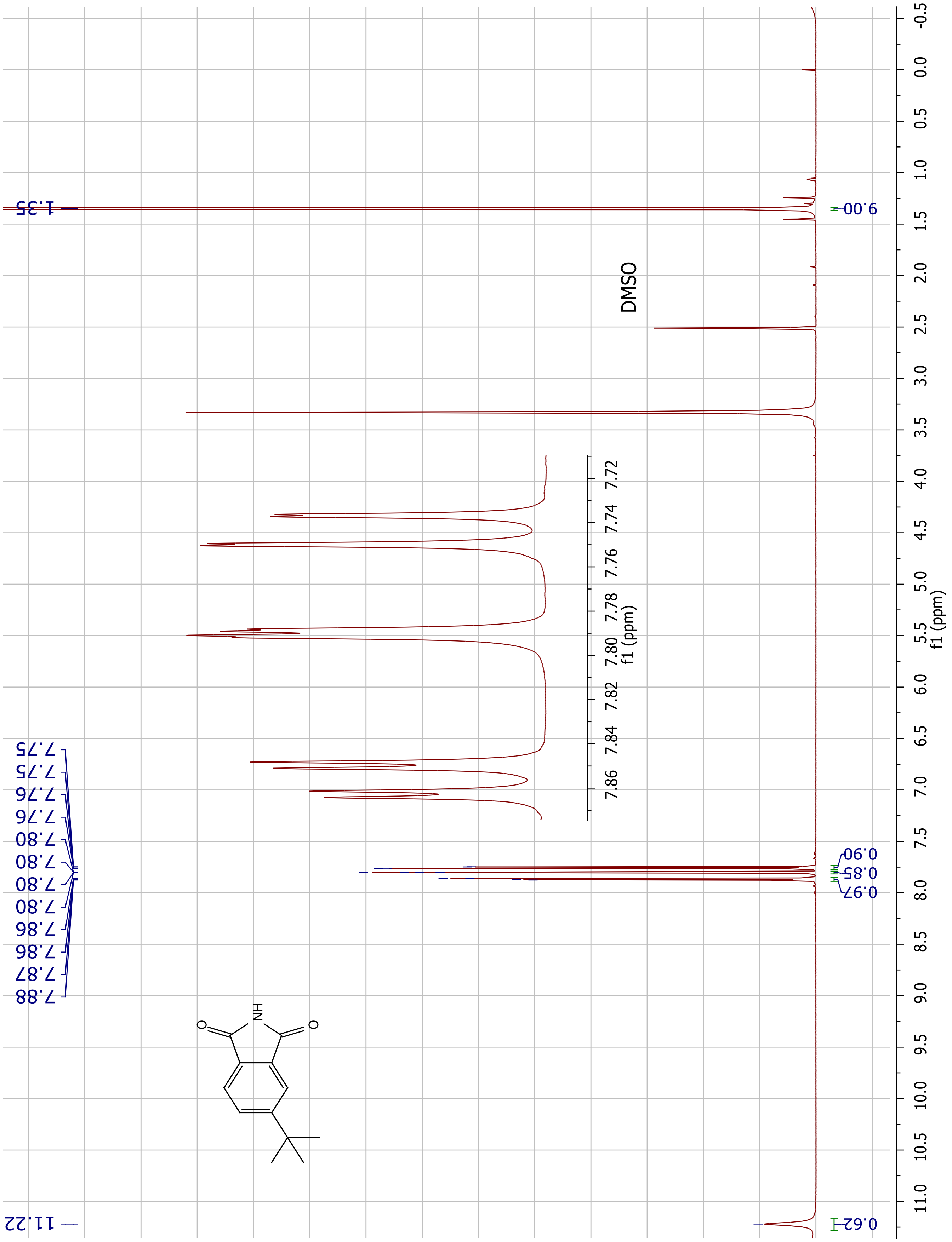

Figure S6a: t-Bu$_4$CoPc ESI-MS.

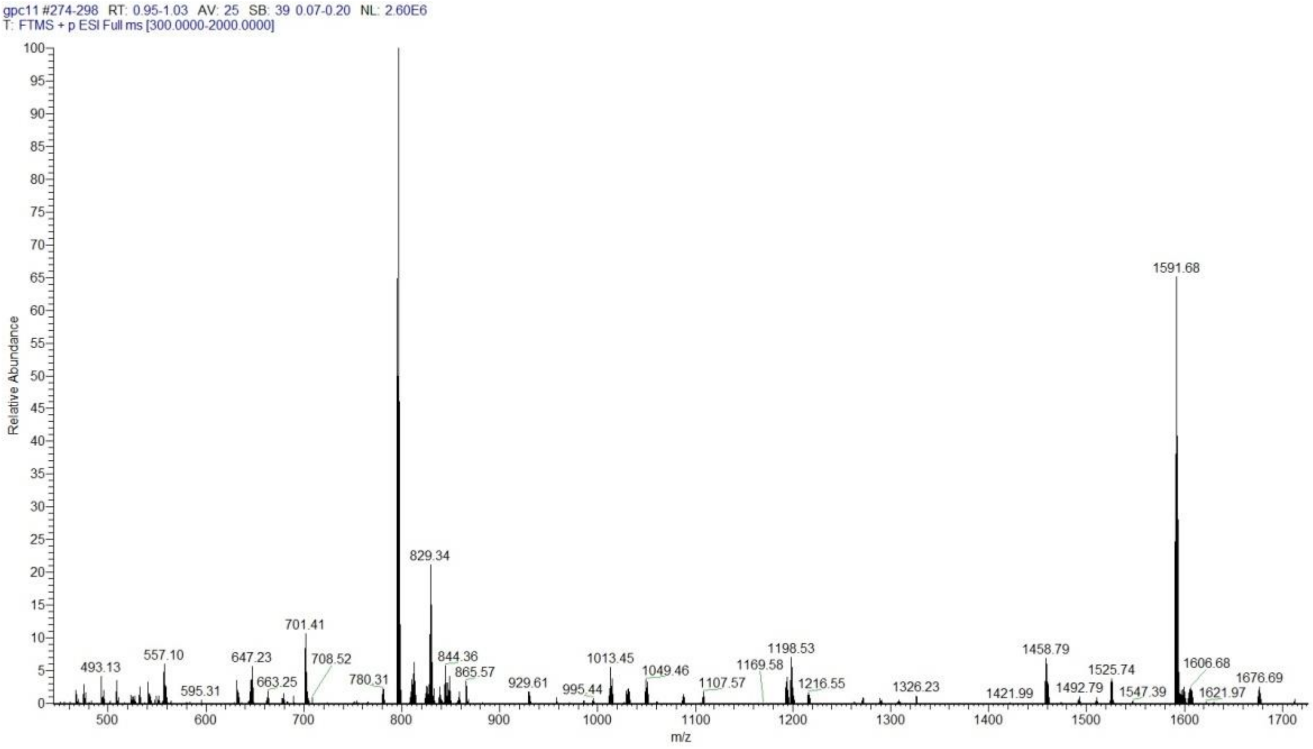

Figure S6b: Magnification of isotopic cluster.

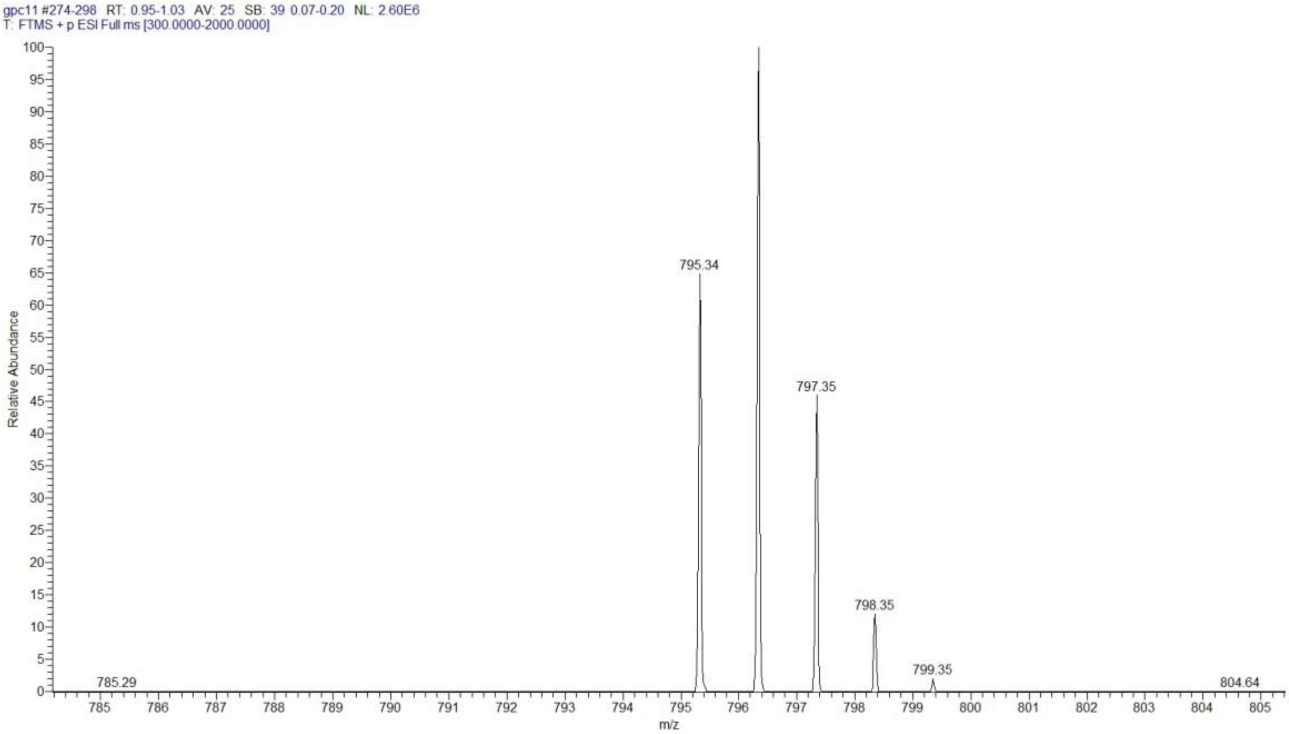

Figure S7a: t-Bu_4CuPc ESI-MS.

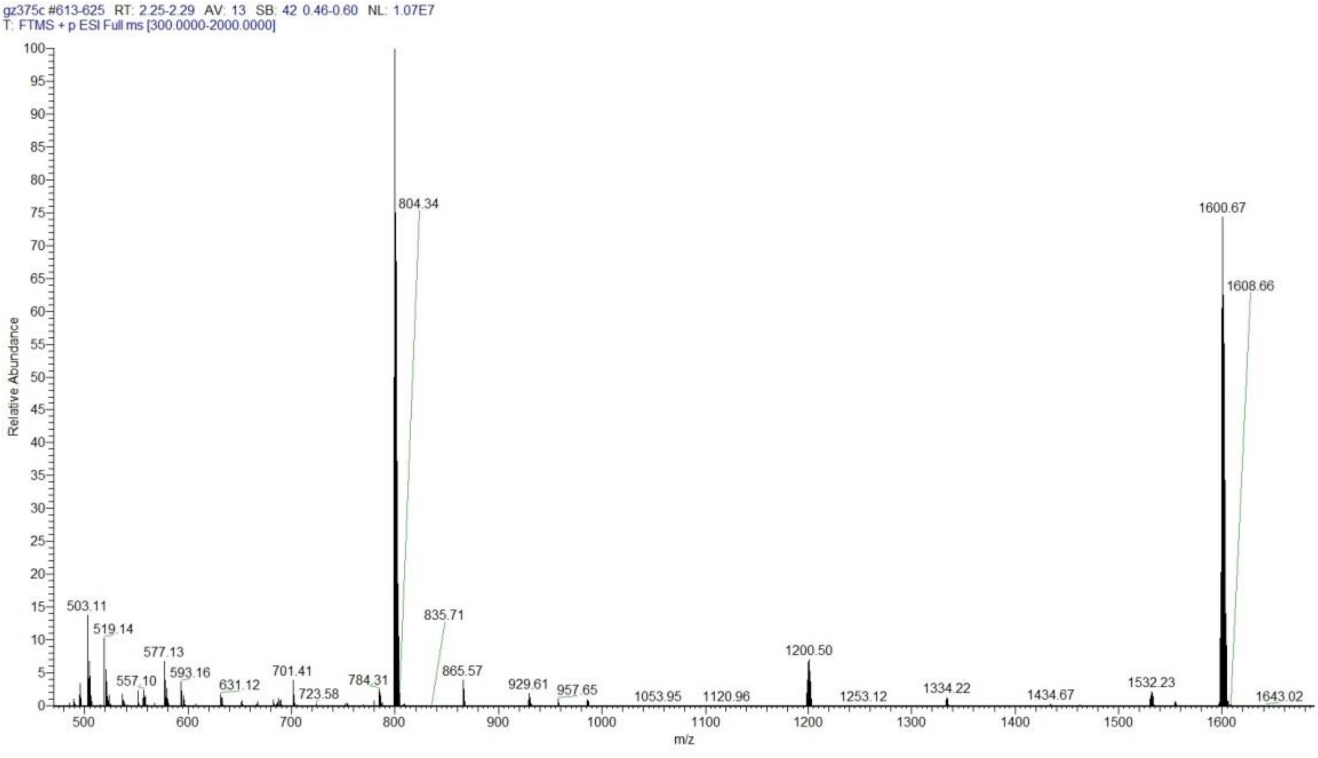

Figure S7b: Magnification of isotopic cluster.

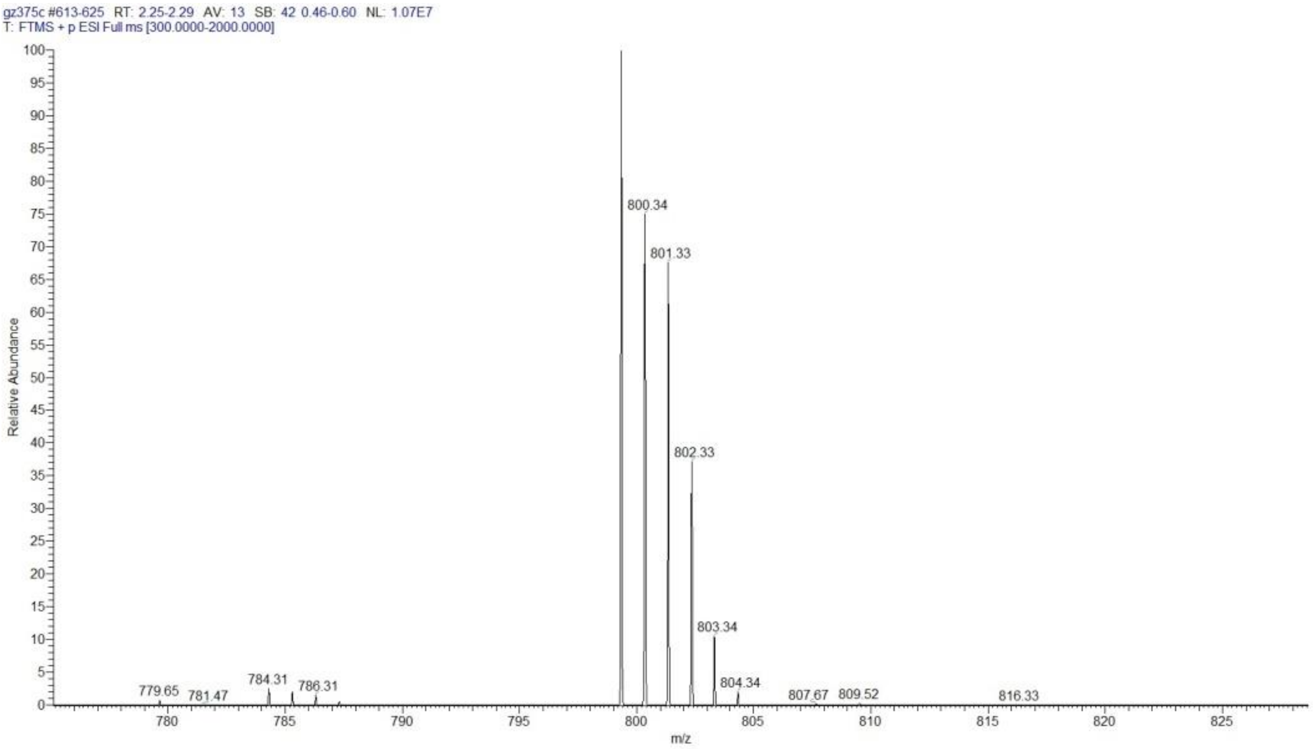

Figure S8a: t-Bu_4ZnPc ESI-MS.

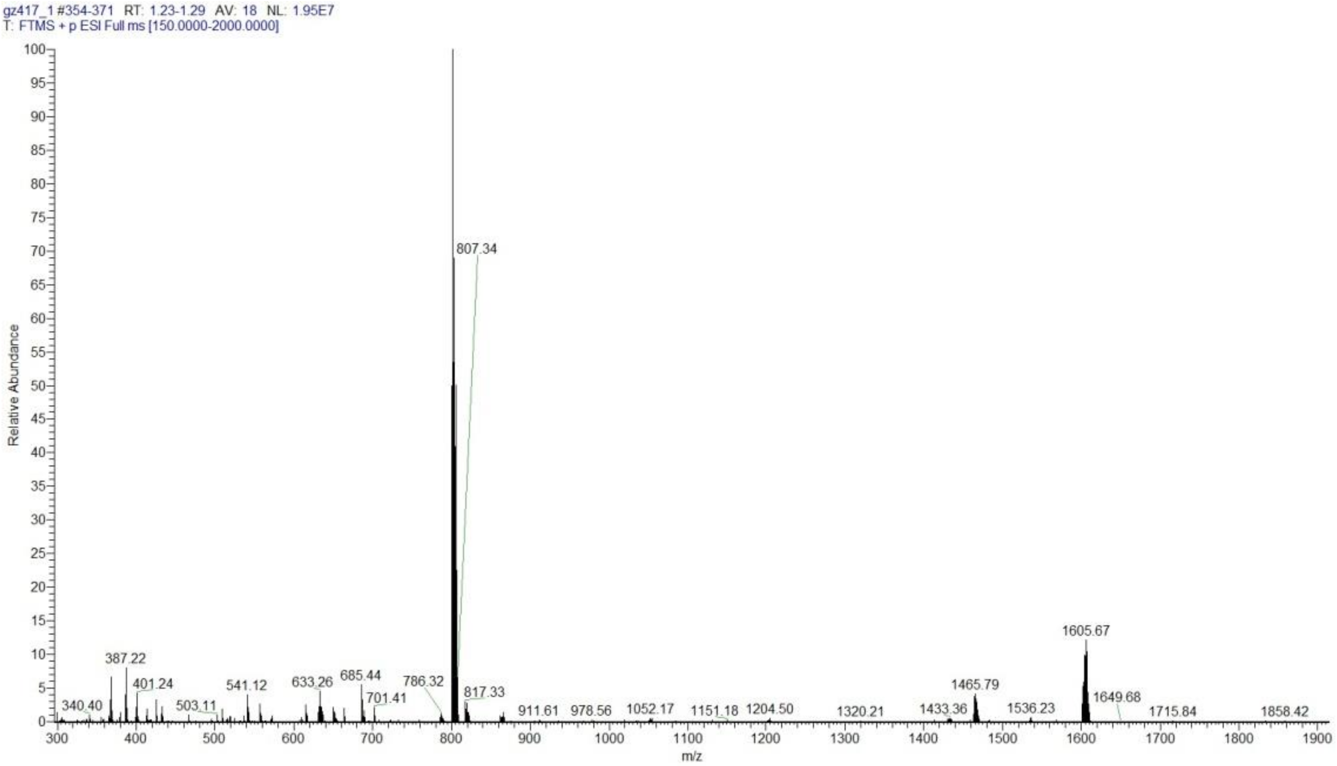

Figure S8b: Magnification of isotopic cluster.

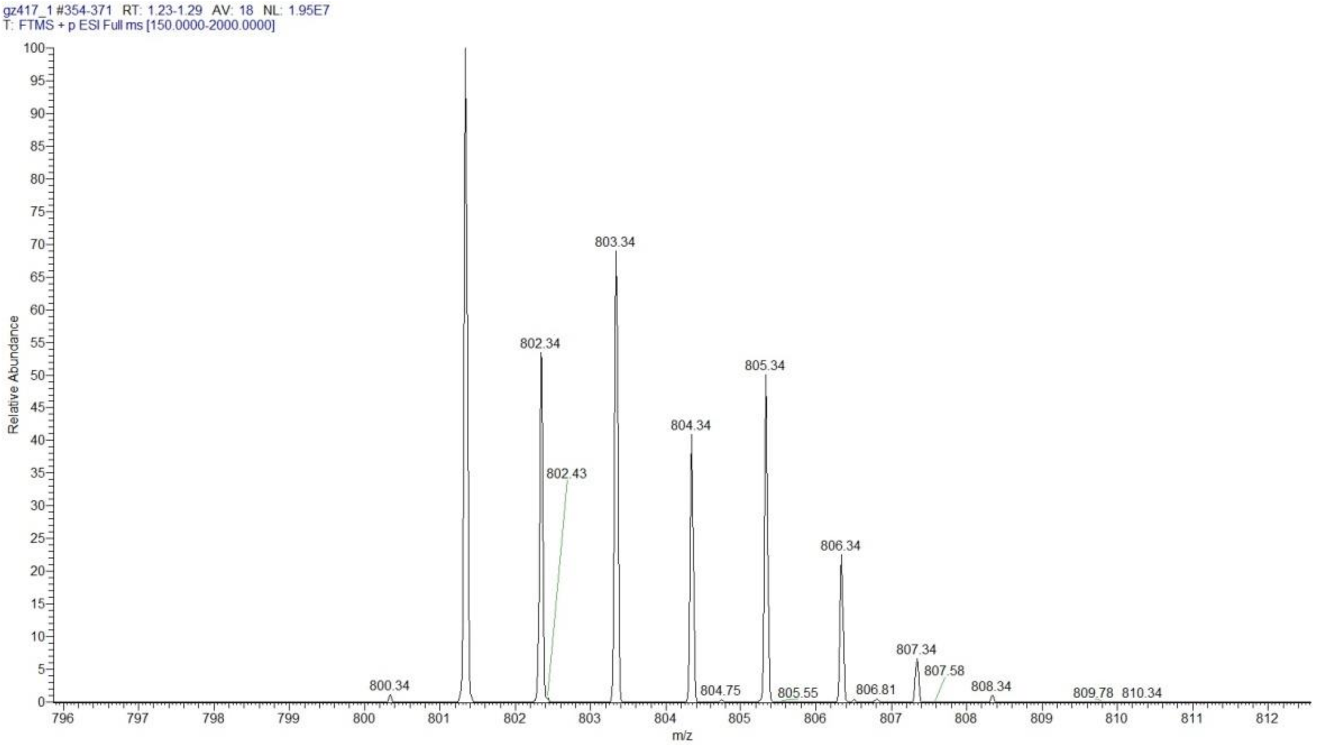

Figure S9: *t*-Bu_3IZnPc ESI-MS.

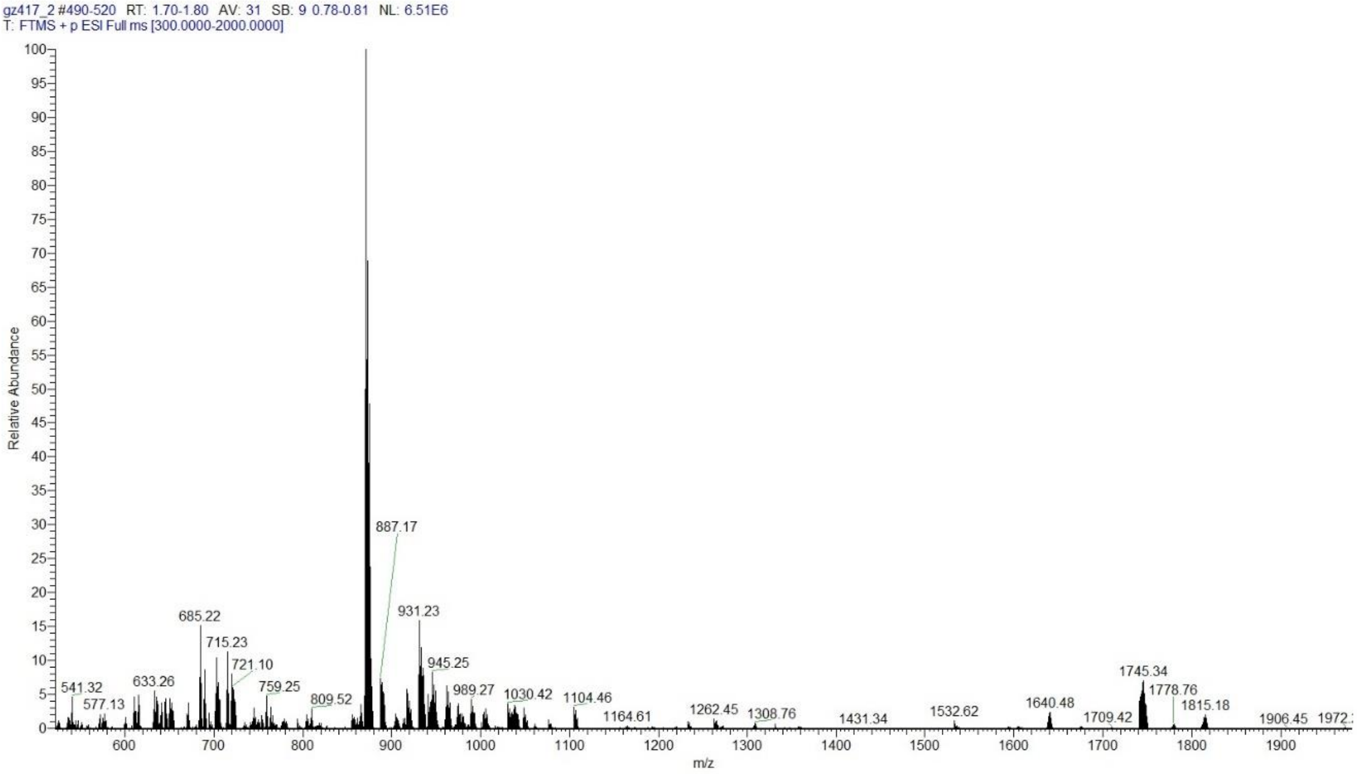

Figure S9b: Magnification of isotopic cluster.

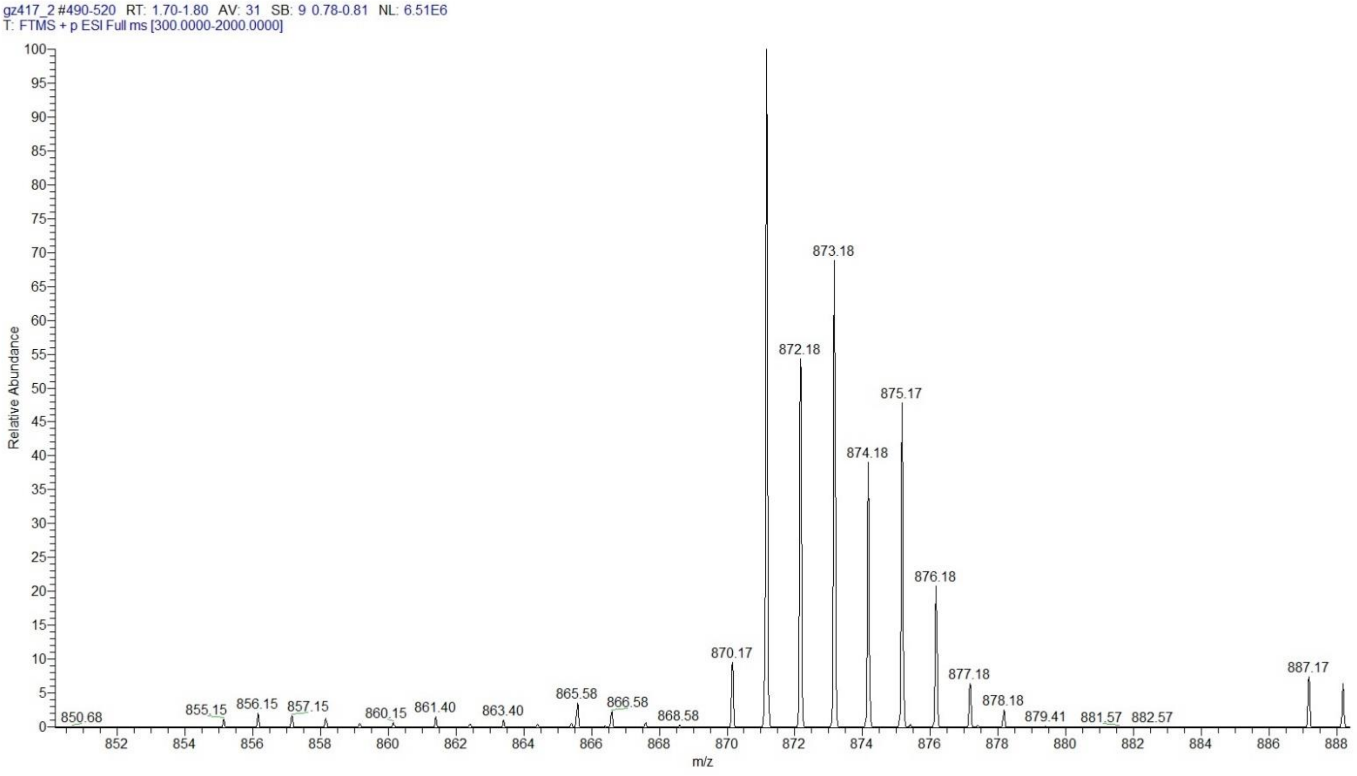

Figure S10a: UV-Vis of CoPc and ZnPc in THF.

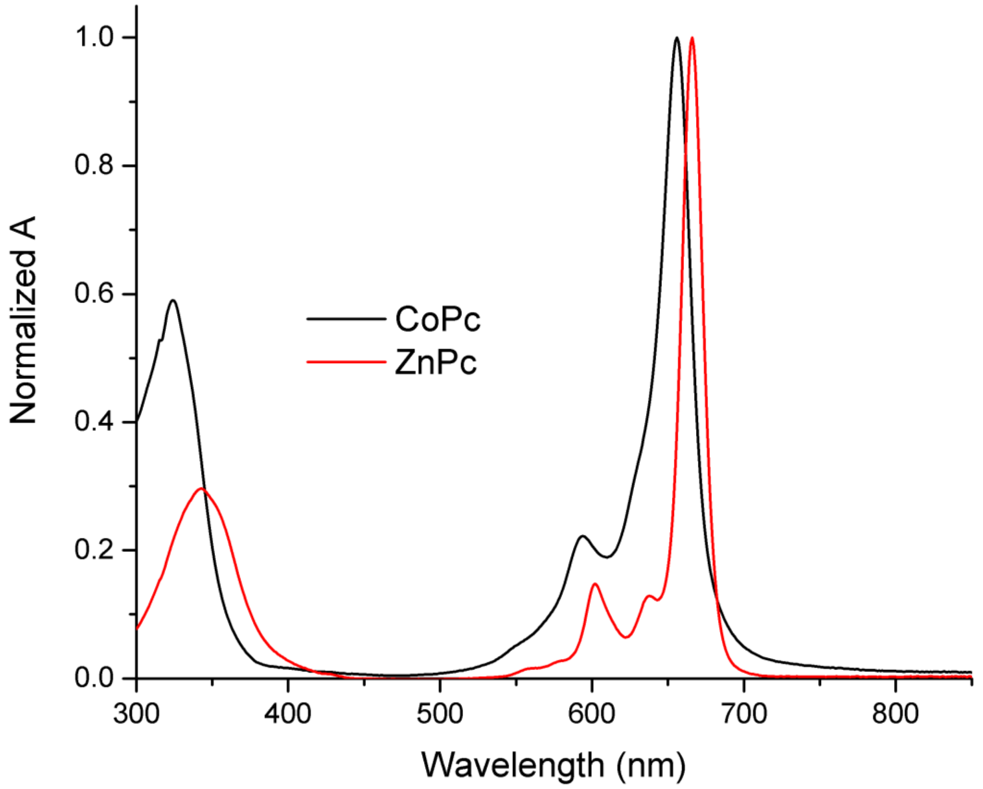

Figure S10b: UV-Vis of CuPc in THF.

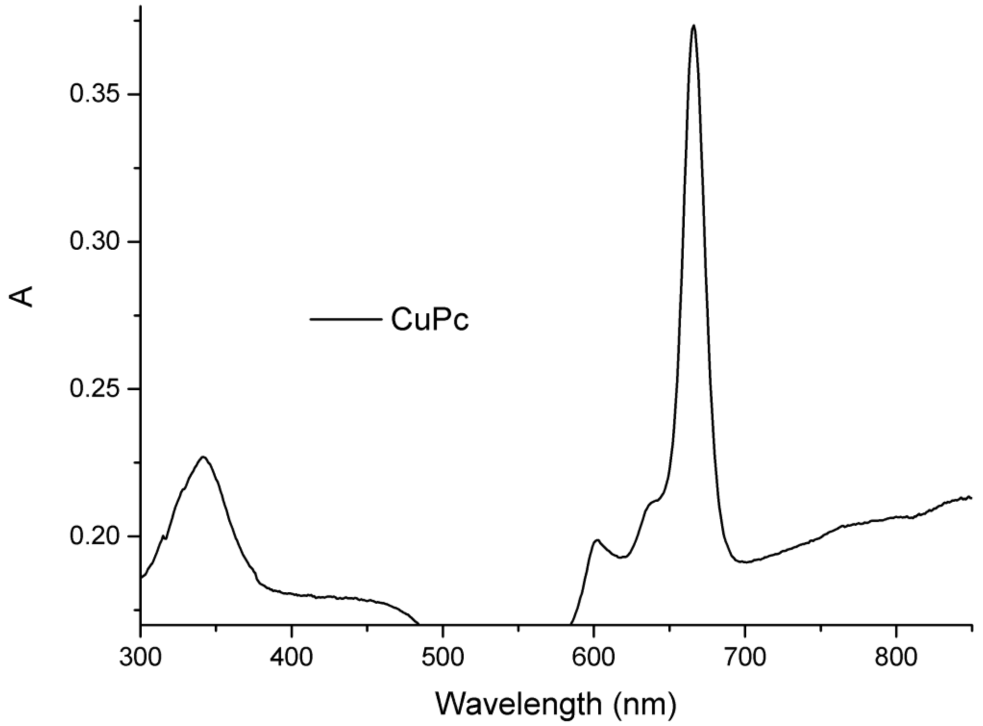

Figure S11: UV-Vis of (*t*-Bu)$_4$substituted phthalocyanines in THF.

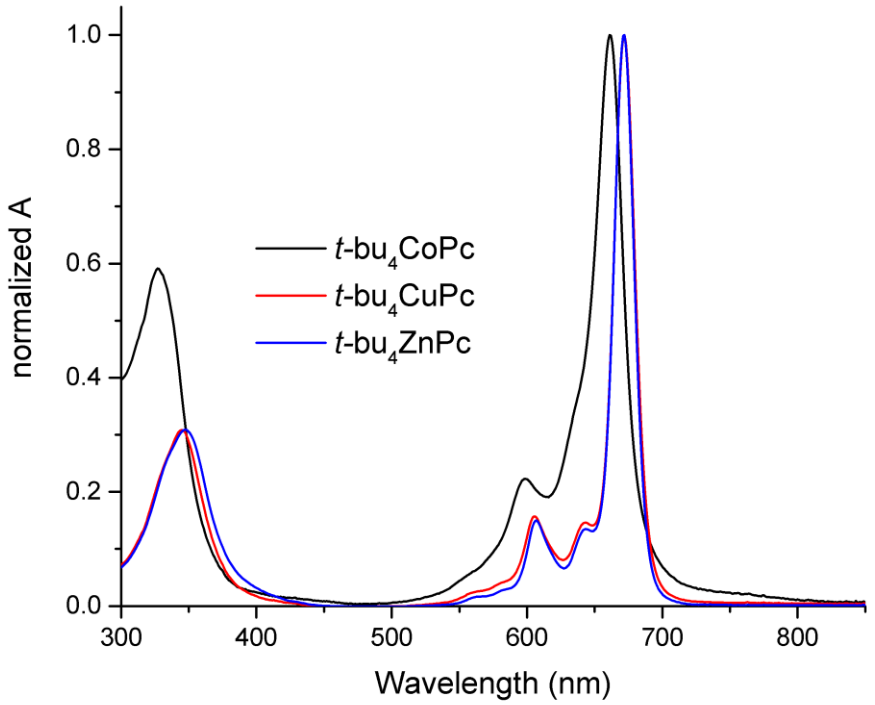

Figure S12: UV-Vis of *t*-bu$_3$IZnPc in THF.

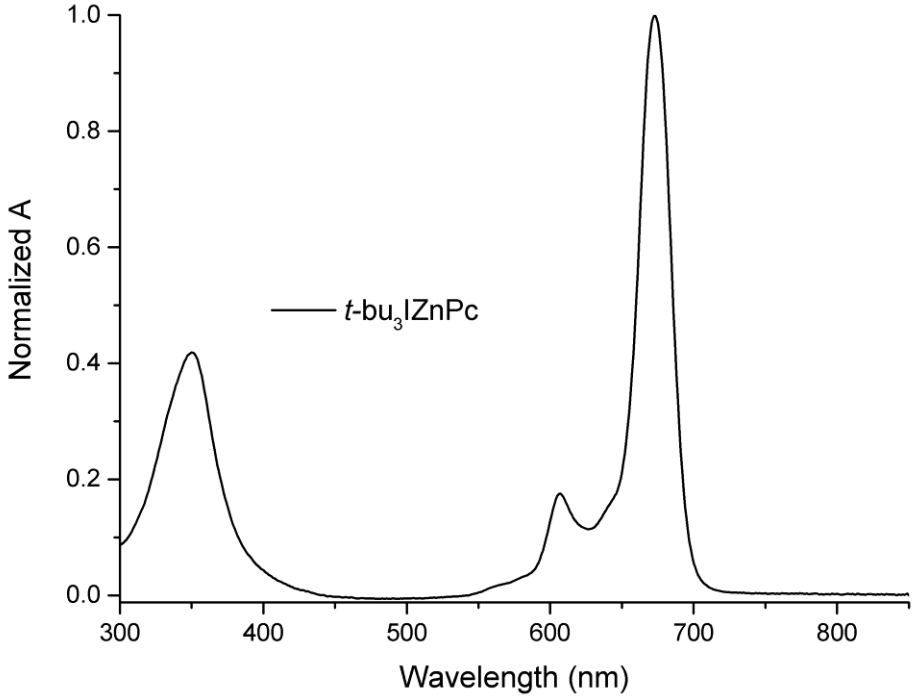

Figure S13: IR spectrum of CoPc.

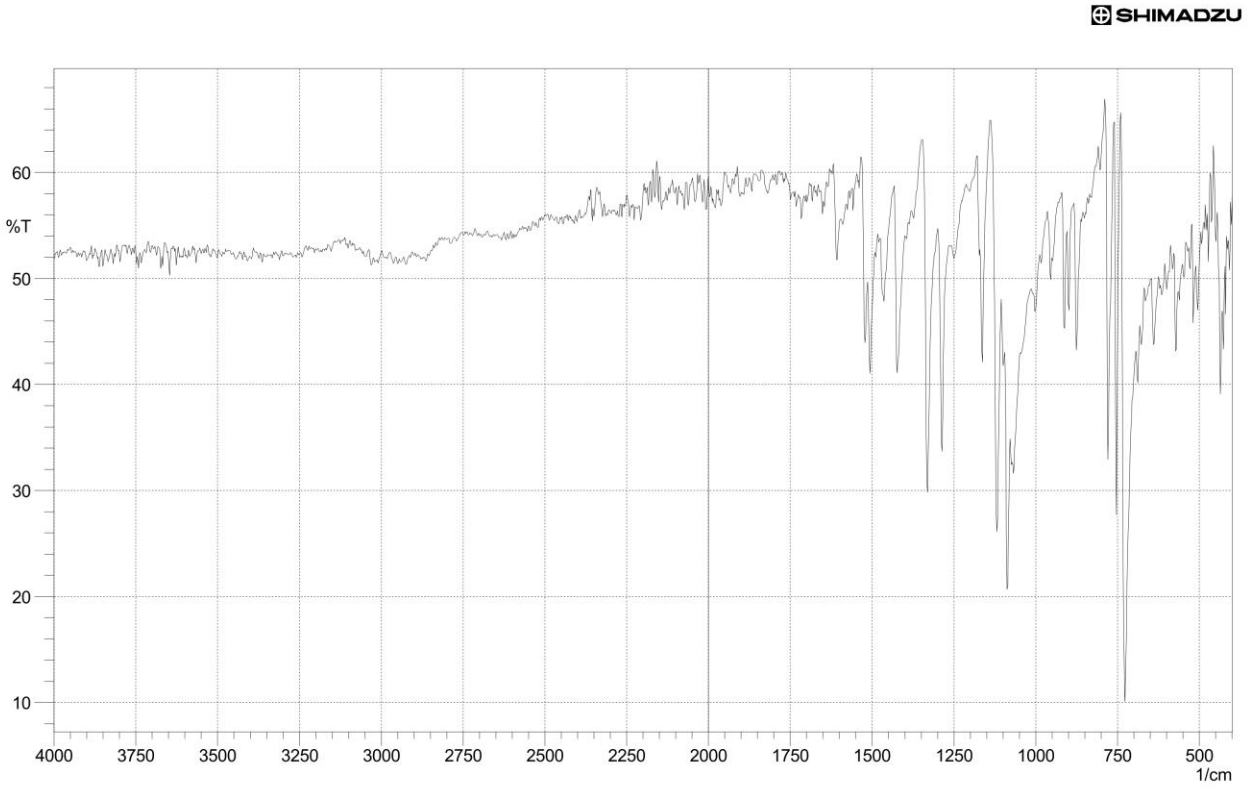

Figure S14: IR spectrum of CuPc.

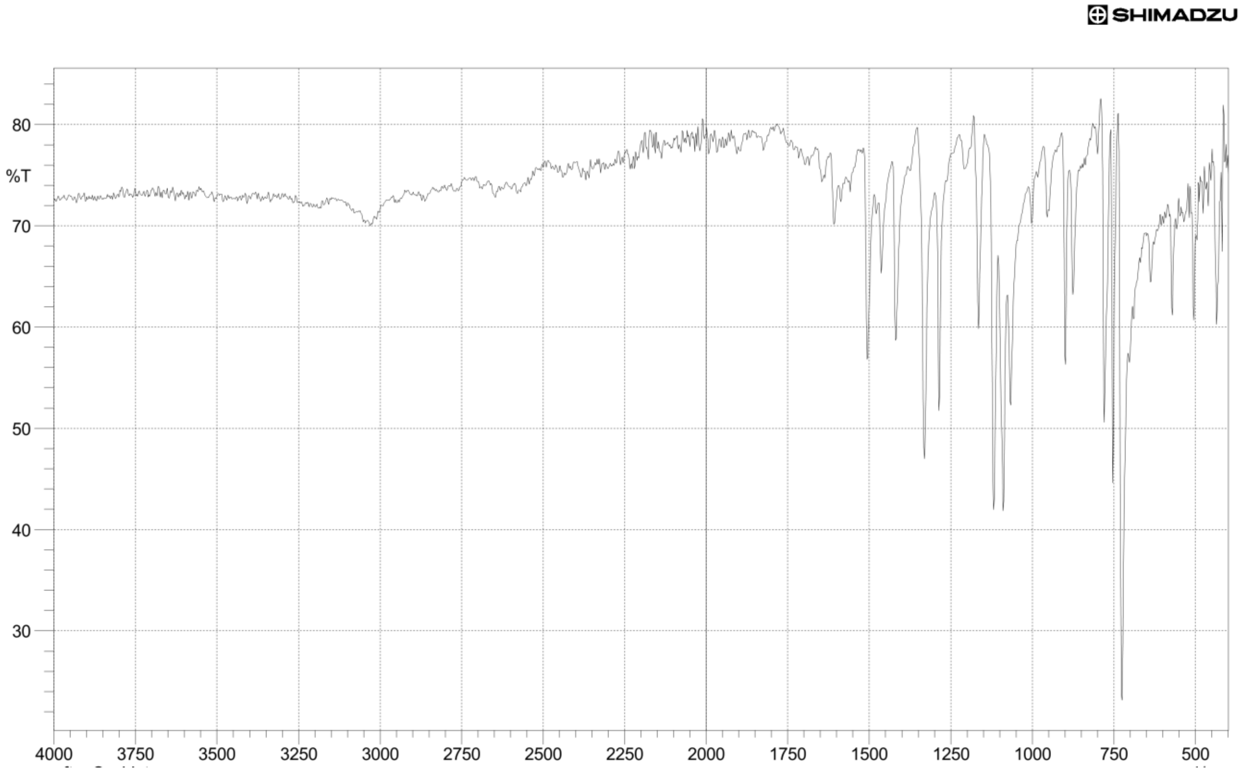

Figure S15: IR spectrum of ZnPc.

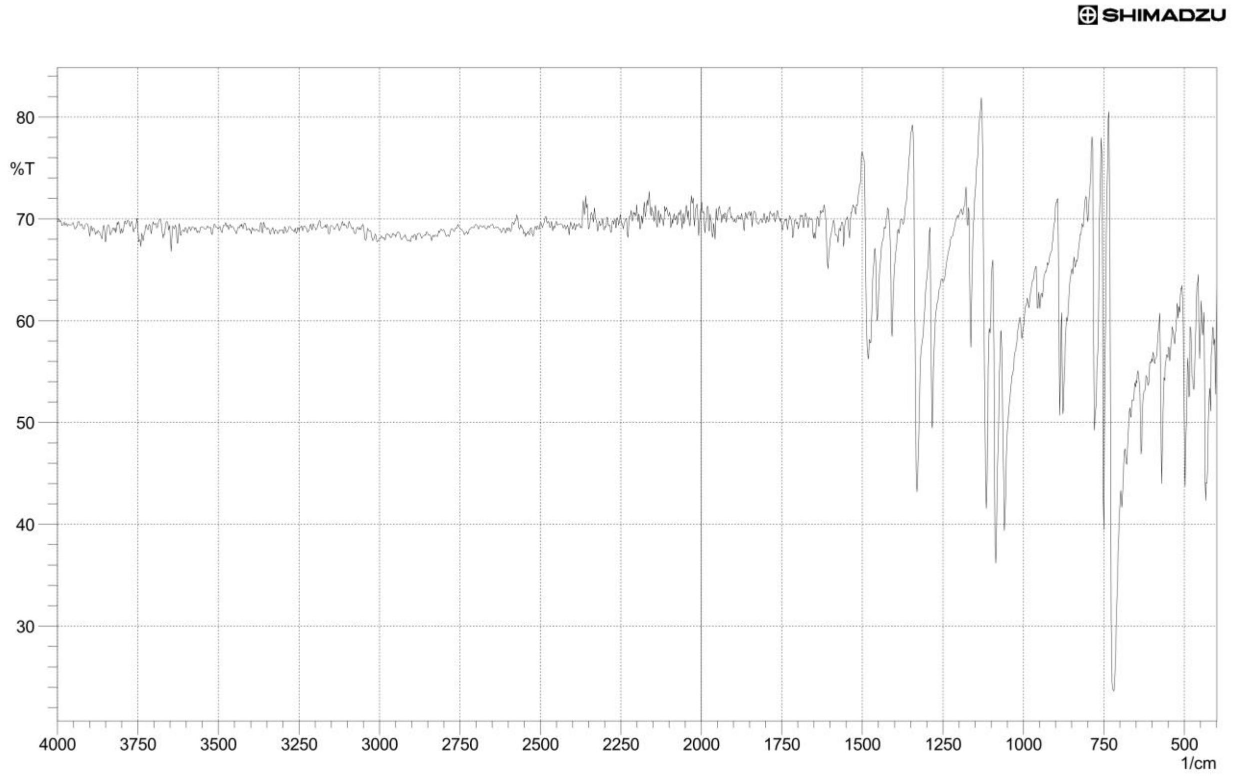

Figure S16: IR spectrum of *t*-Bu_4CoPc.

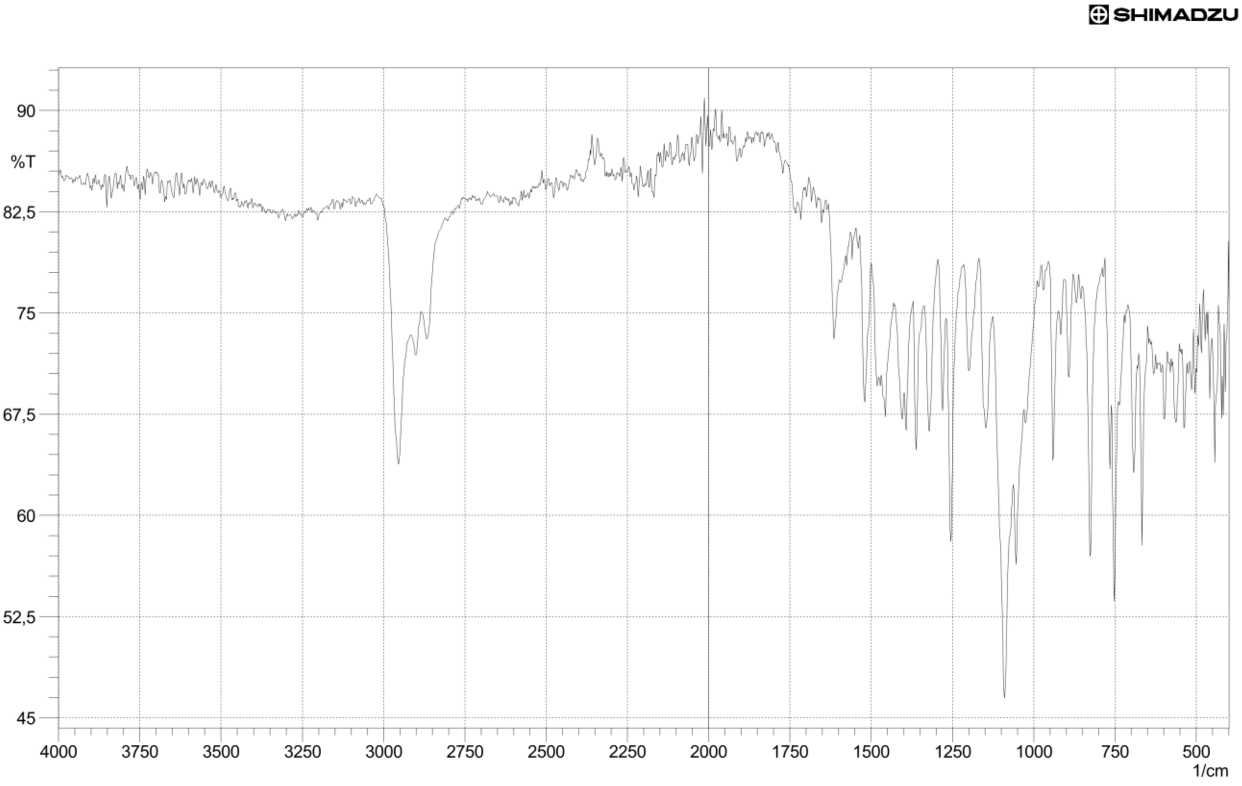

Figure S17: IR spectrum of *t*-Bu_4CuPc.

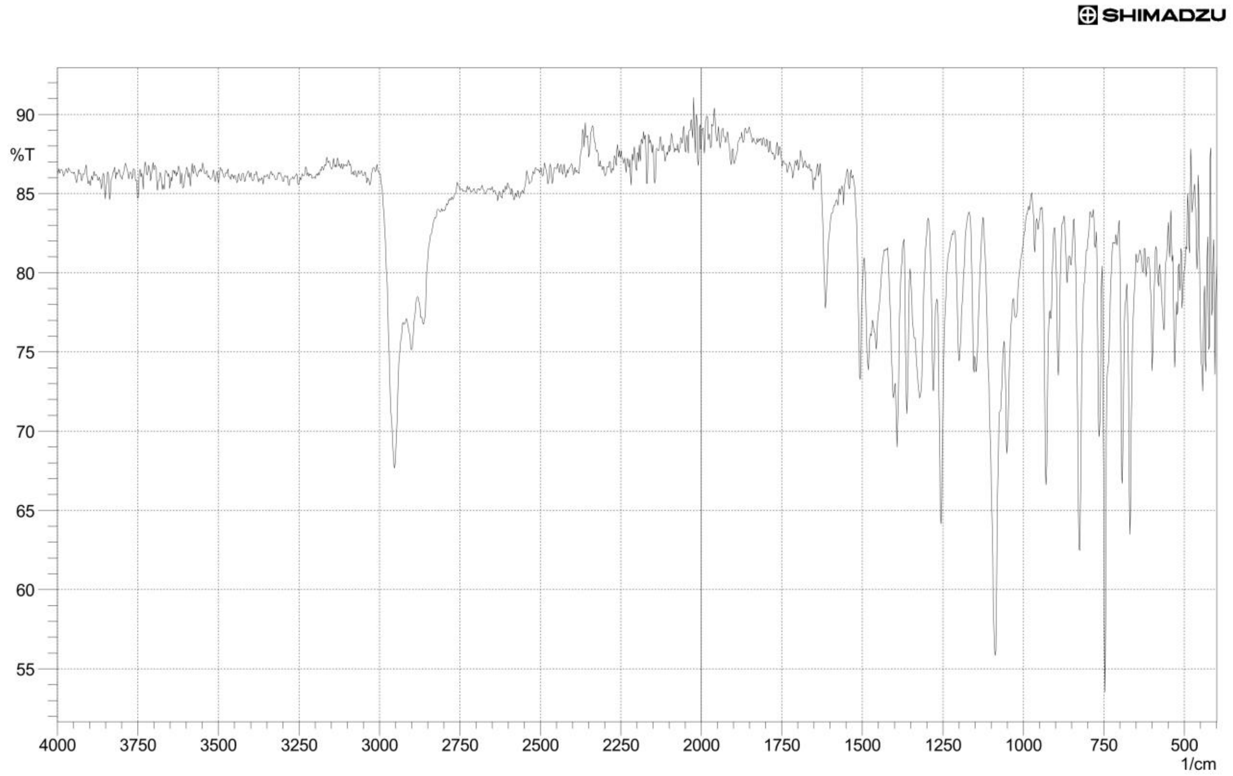

Figure S18: IR spectrum of *t*-Bu_4ZnPc.

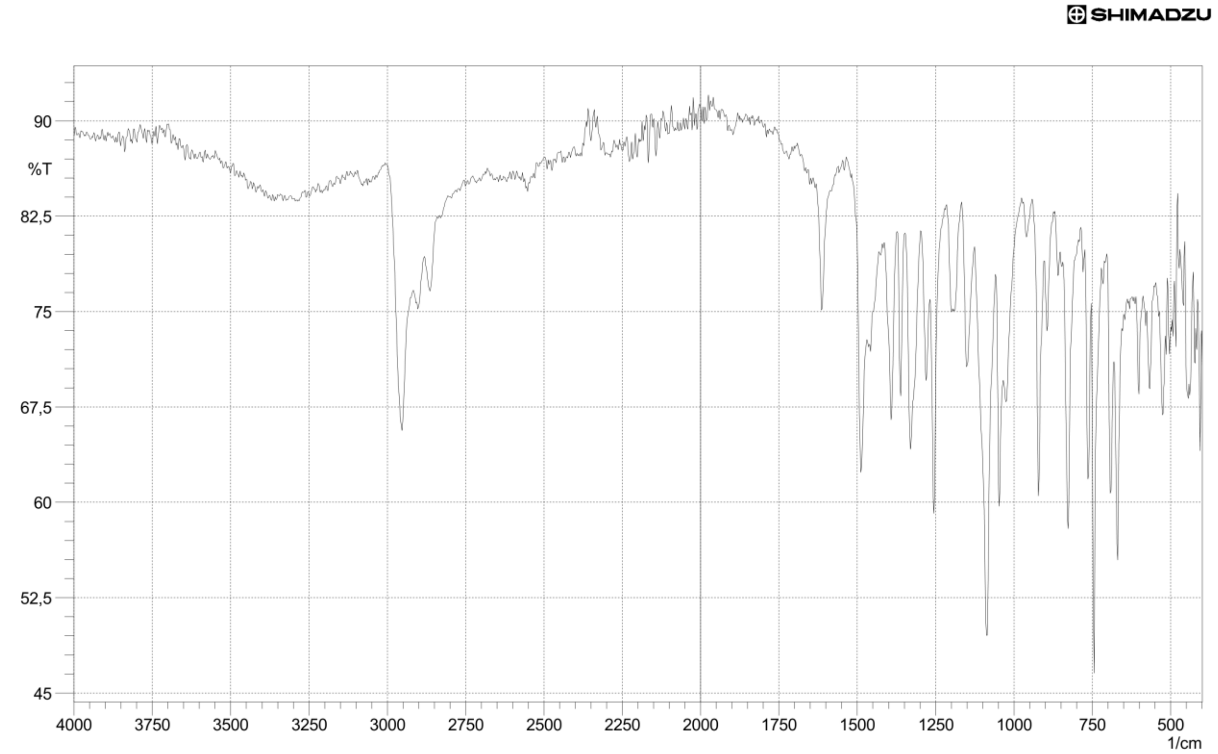

Figure S19: IR spectrum of t-Bu$_3$IZnPc.

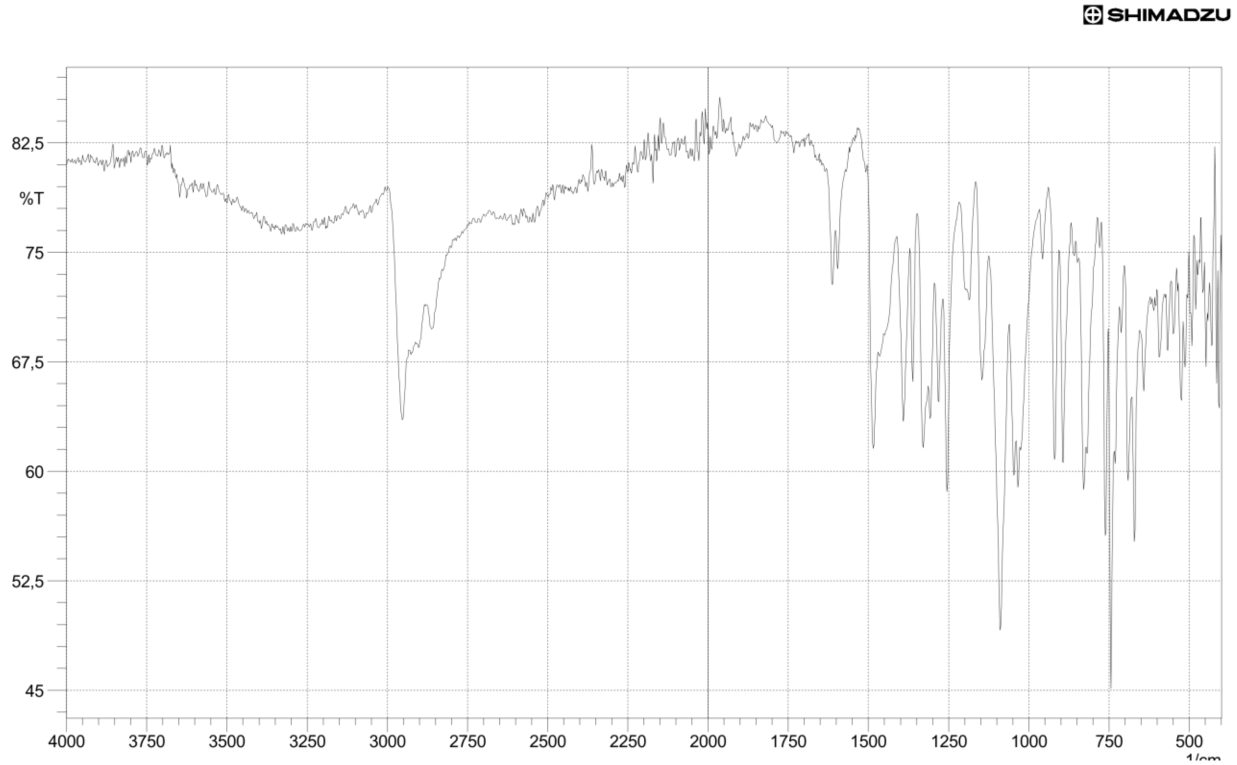

Cost analysis

Merck has been chosen as the only supplier for sake of simplicity, calculations have been made with prices at Jan 31st, 2021. The biggest batch available without inquiring a bulk quotation has been chosen, at a sufficient degree of purity. The related SKU codes have been reported for sake of clarity.
The purification step with acetic acid in the Soxhlet apparatus has not been included in the cost analysis, because the solvent is recyclable many times.
The amount of waste is inclusive of the excess of salt that we used.
The density of HCl 1N has been approximated to 1.00 g/cm^3.

Table S1: Overview of the quoted materials

	quoted price (EUR)	size	EUR/kg
Phthalonitrile<br>8005939050	2130.00	50 kg	42.60
4-*tert*-butylphthalonitrile<br>423122	102.00	1g	102000.00
$Co(OAc)_2{\cdot}4H_2O$<br>208396-1KG	378.00	1000 g	378.00
$Cu(OAc)_2{\cdot}H_2O$<br>341746-2.5KG	339.00	2.5 kg	135.60
$Zn(OAc)_2{\cdot}2H_2O$<br>96459-1KG	134 .00	1 kg	134.00
DBU<br>8032829050<br>d= 1.02 g/cm^3	2,350.00	50 kg	47.00
KOH “for synthesis”<br>8143535000	160.00	5 kg	32.00
DMAE “>99.5%”<br>890 kg/m^3 (0.890 g/cm^3)<br>471453-2L	84.00	2l<br>1.780 g	47.20
Anisole “for synthesis”<br>8014529025<br>d= 0.995 g/cm^3	670.00	25 l<br>24875 g	26.90
Glycerol “anhydrous for synthesis”<br>8187099025<br>d= 1.26 g/cm^3	527.00	25 l<br>31.5 kg	16.70
Methanol “Laboratory Reagent, ≥99.6%”<br>179957-25L<br>d= 0.791 g/cm^3	266.00	25 l<br>19.77 kg	13.40
HCl “puriss. p.a., ACS reagent, reag. ISO, reag. Ph. Eur., fuming, ≥37%, APHA: ≤10”<br>30721-2.5L-M<br>d= 1.2 g/cm^3	40.50	2.5 l<br>3.0 kg	13.50
Cobalt phthalocyanine β-form, Dye content 97 %<br>307696-50G	527.00	50 g	10540.00
Copper phthalocyanine β-form, Dye content 90 %<br>252980-100G	152.00	100 g	1520.00
Zinc phthalocyanine Dye content 97 %<br>341169-25G	147.00	25 g	5880.00

Figure S20: Flowchart for the synthesis of 1 kg of CoPc in standard conditions**.** Reagents quantities are highlighted in red, solvent is highlighted in green and workup/purification materials are highlighted in blue.

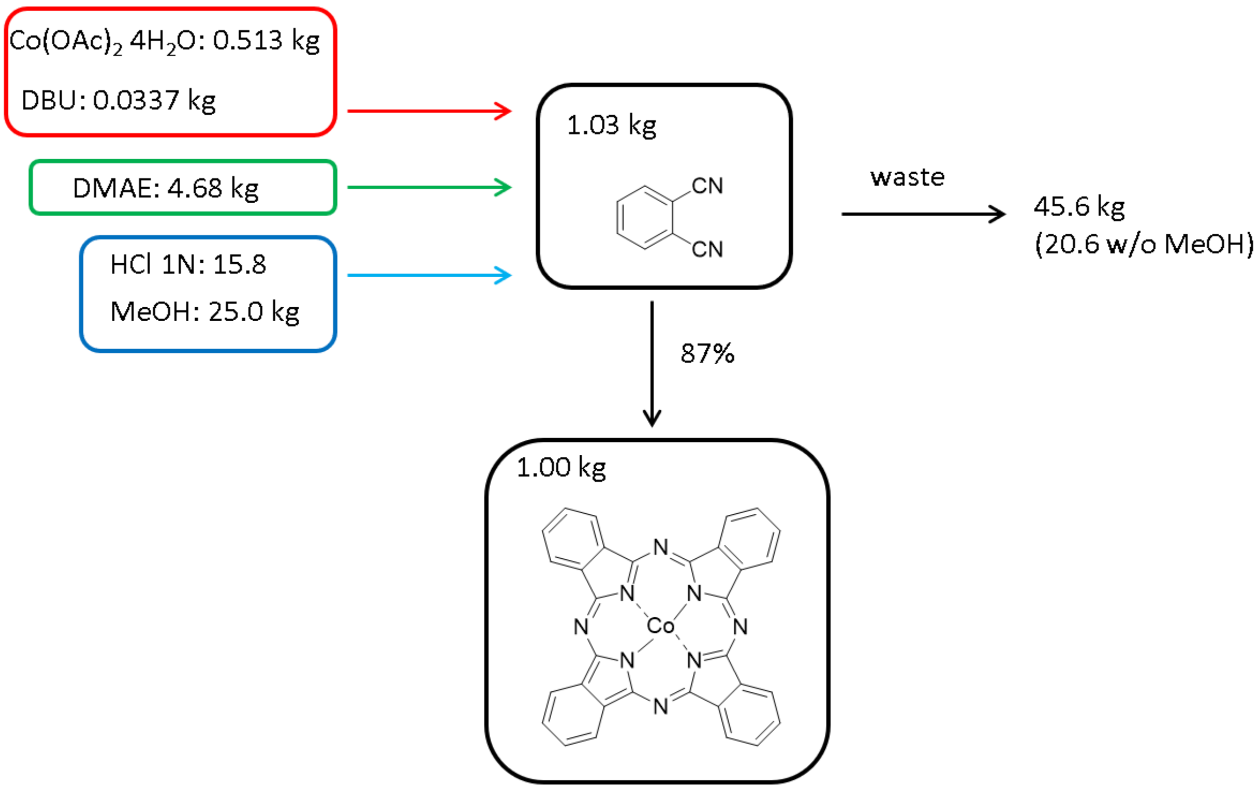

Figure S21: Flowchart for the synthesis of 1 kg of CuPc in standard conditions**.** Reagents quantities are highlighted in red, solvent is highlighted in green and workup/purification materials are highlighted in blue.

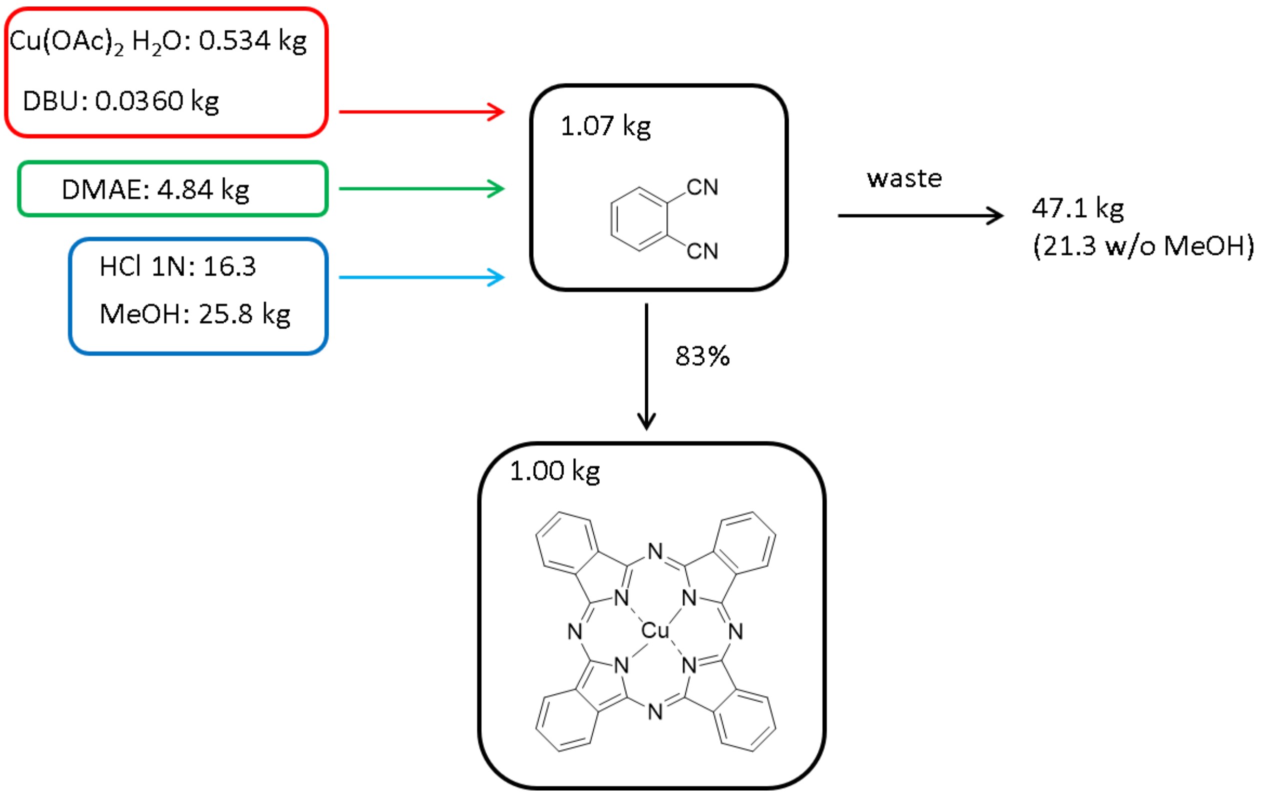

Figure S22: Flowchart for the synthesis of 1 kg of ZnPc in standard conditions**.** Reagents quantities are highlighted in red, solvent is highlighted in green and workup/purification materials are highlighted in blue.

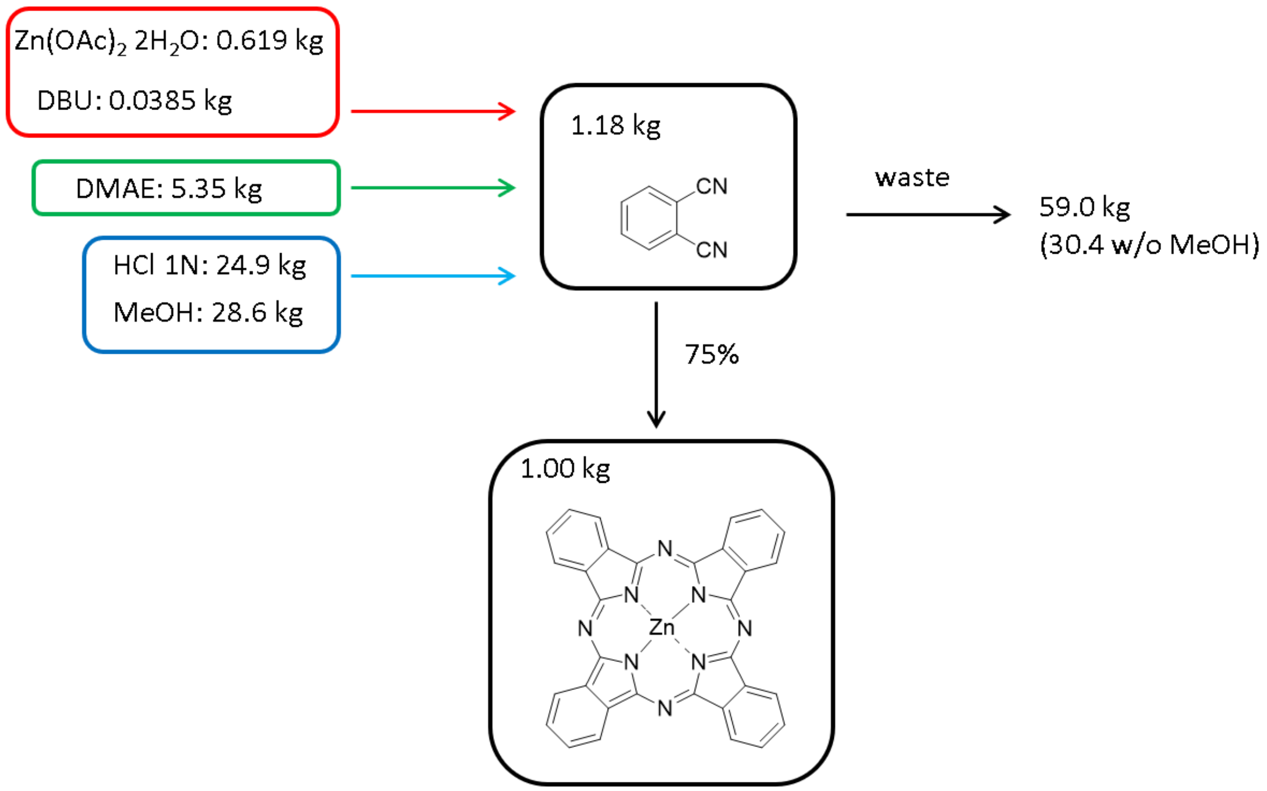

Figure S23: Flowchart for the synthesis of 1 kg of CoPc in A-DBU conditions. Reagents quantities are highlighted in red, solvent is highlighted in green and workup/purification materials are highlighted in blue.

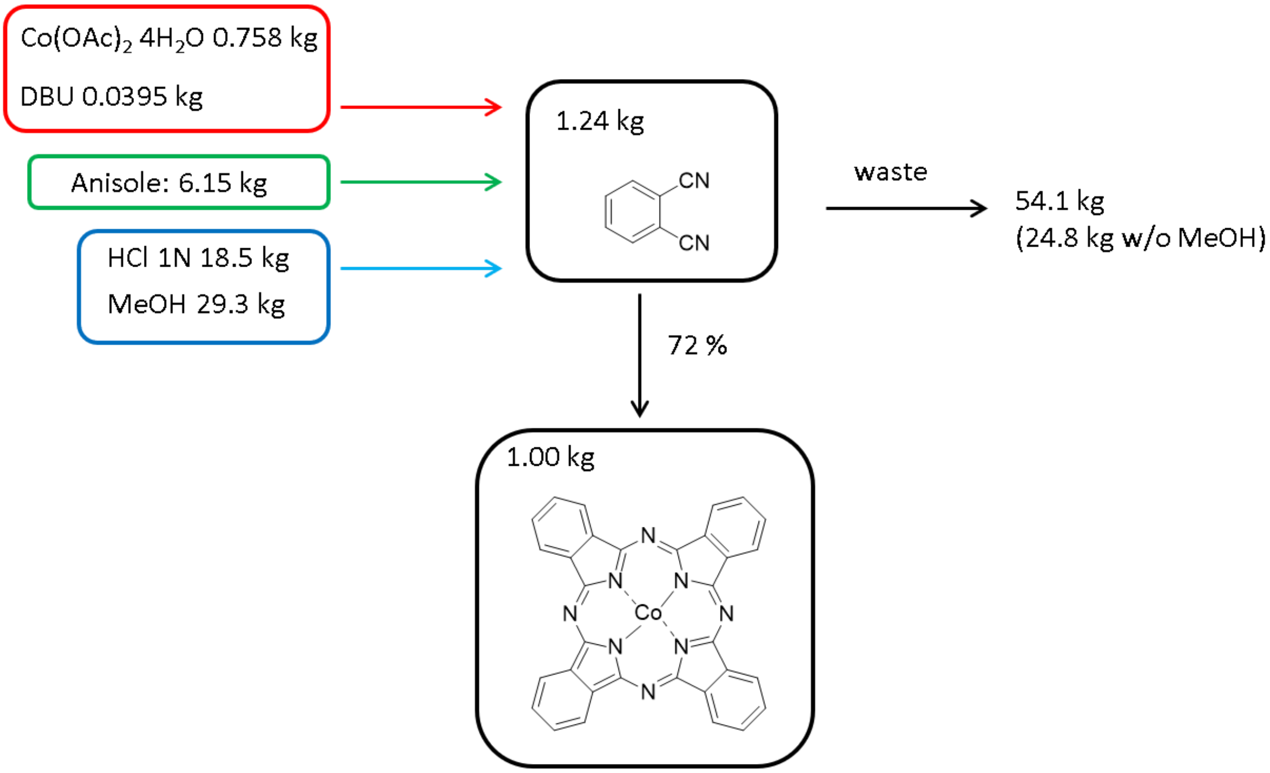

Figure S24: Flowchart for the synthesis of 1 kg of CuPc in A-DBU conditions**.** Reagents quantities are highlighted in red, solvent is highlighted in green and workup/purification materials are highlighted in blue.

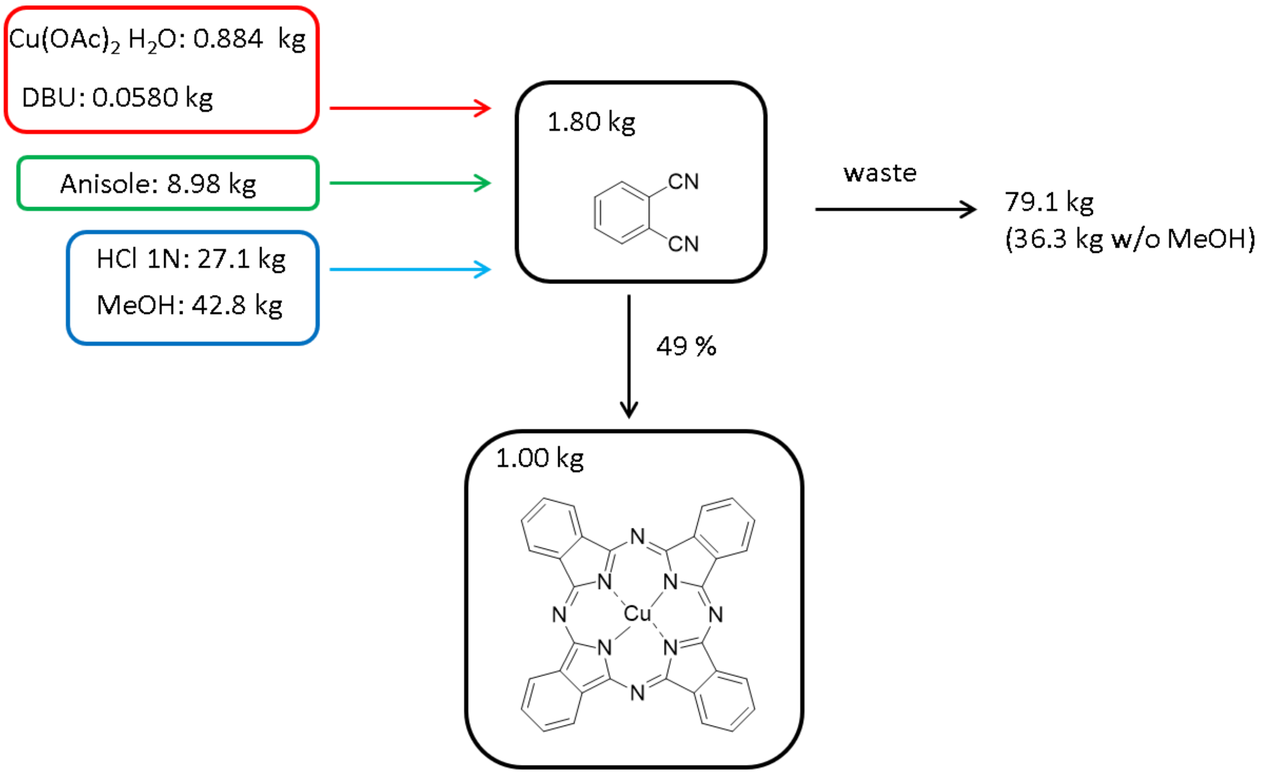

Figure S25: Flowchart for the synthesis of 1 kg of ZnPc in A-DBU conditions. Reagents quantities are highlighted in red, solvent is highlighted in green and workup/purification materials are highlighted in blue.

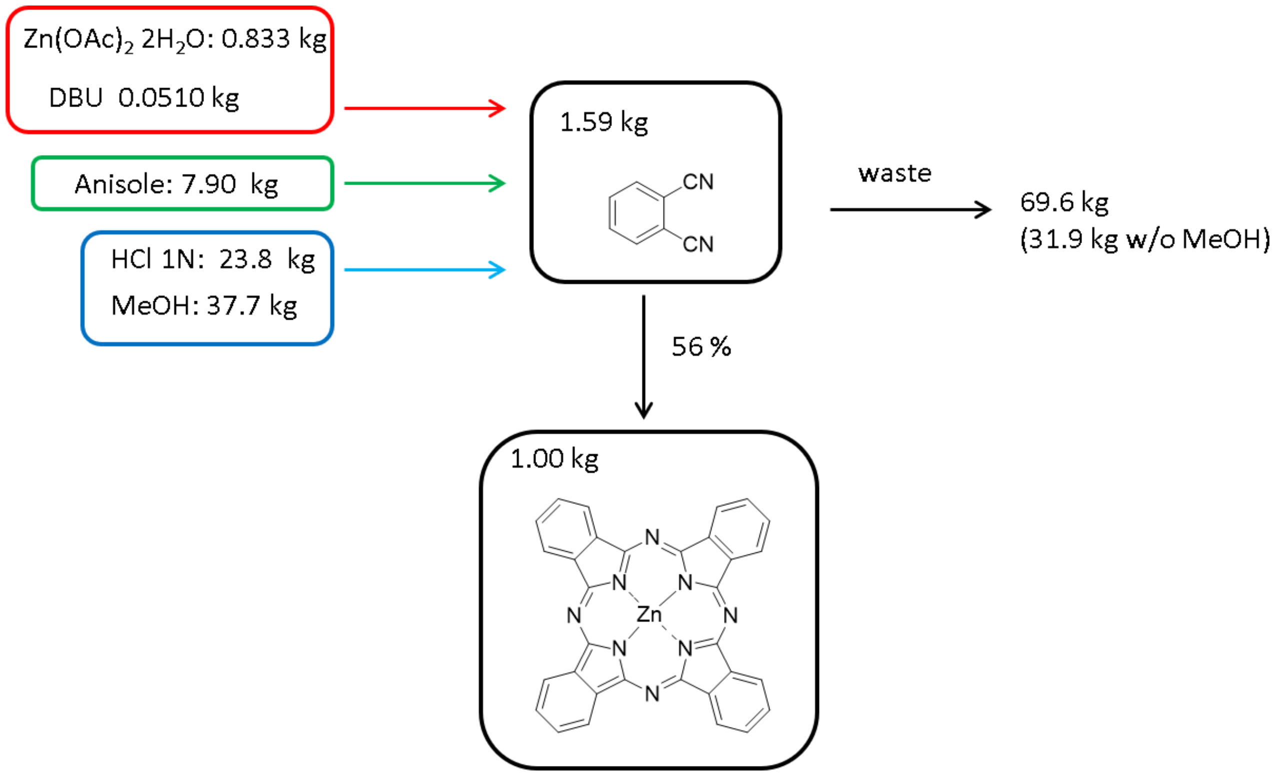

Figure S26: Flowchart for the synthesis of 1 kg of CoPc in A-KOH conditions. Reagents quantities are highlighted in red, solvent is highlighted in green and workup/purification materials are highlighted in blue.

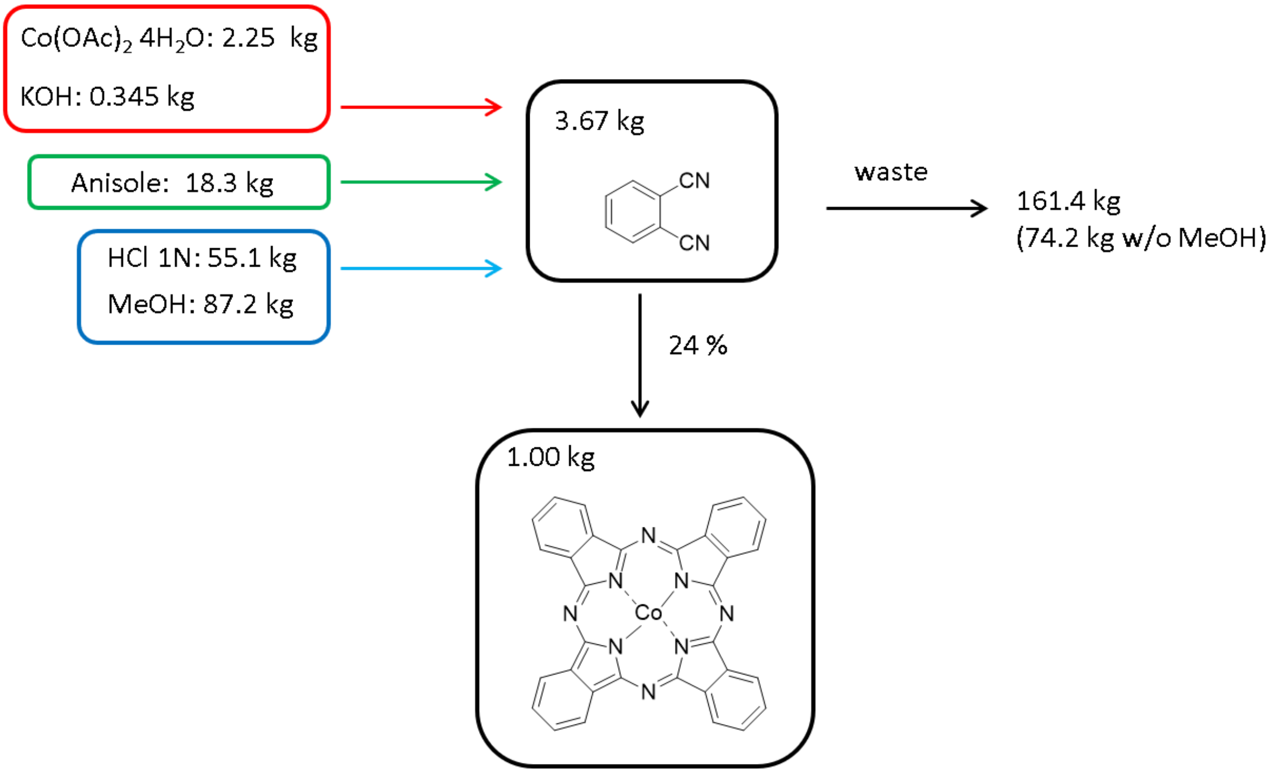

Figure S27: Flowchart for the synthesis of 1 kg of CuPc in A-KOH conditions**.** Reagents quantities are highlighted in red, solvent is highlighted in green and workup/purification materials are highlighted in blue.

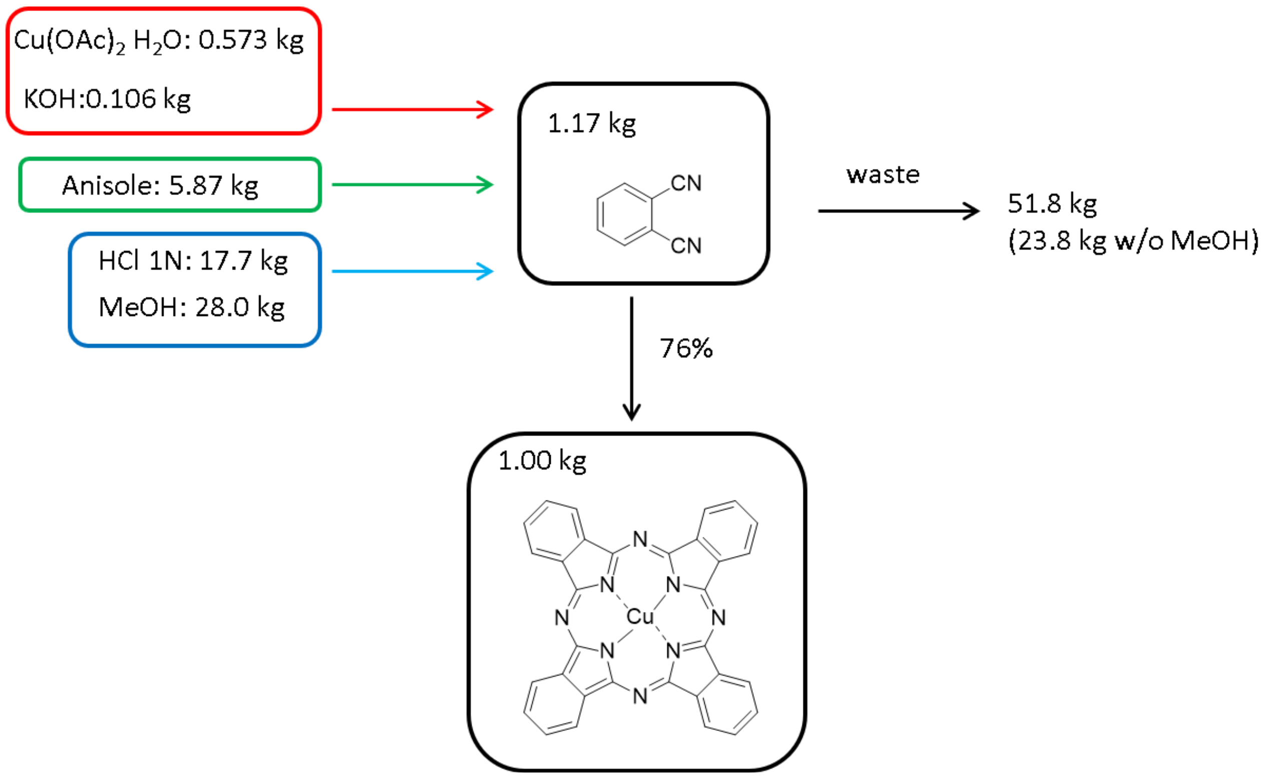

Figure S28: Flowchart for the synthesis of 1 kg of CoPc in GA-KOH conditions. Reagents quantities are highlighted in red, solvent is highlighted in green and workup/purification materials are highlighted in blue.

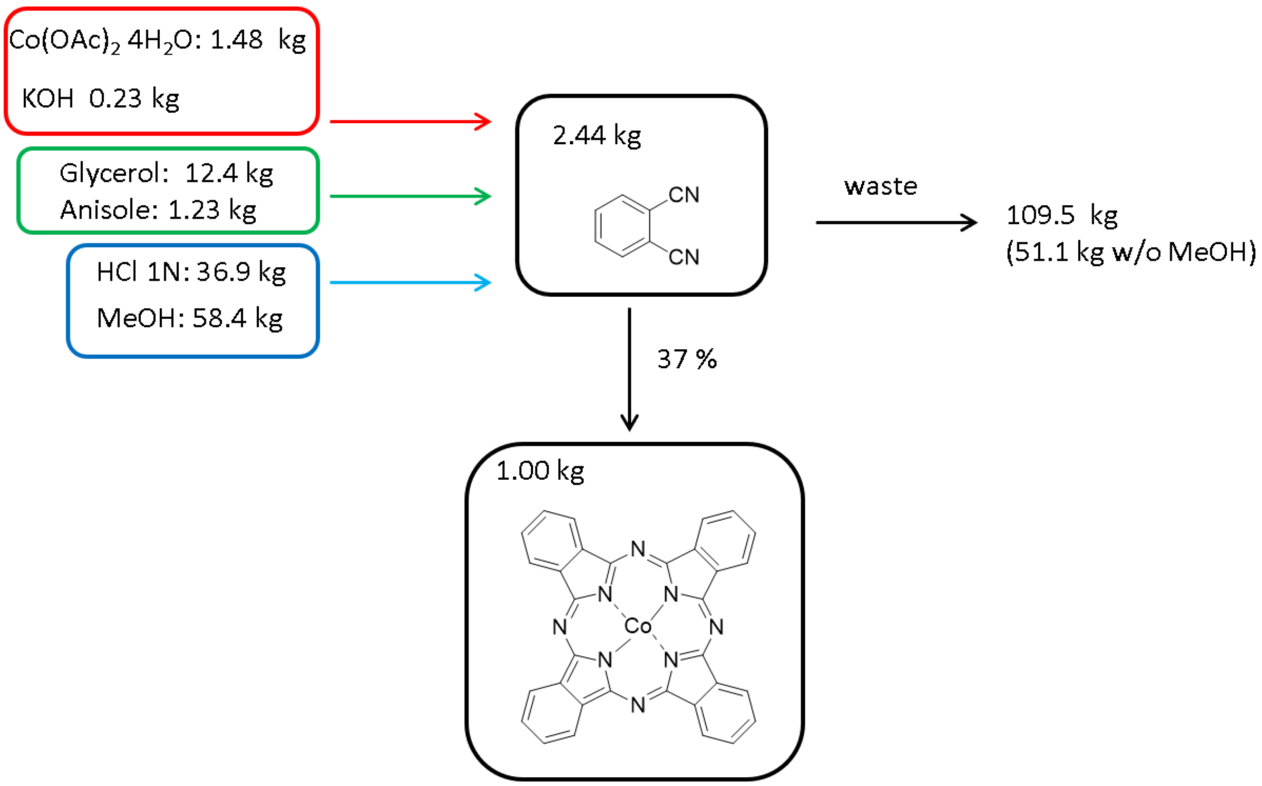

Figure S29: Flowchart for the synthesis of 1 kg of CuPc in GA-KOH conditions. Reagents quantities are highlighted in red, solvent is highlighted in green and workup/purification materials are highlighted in blue.

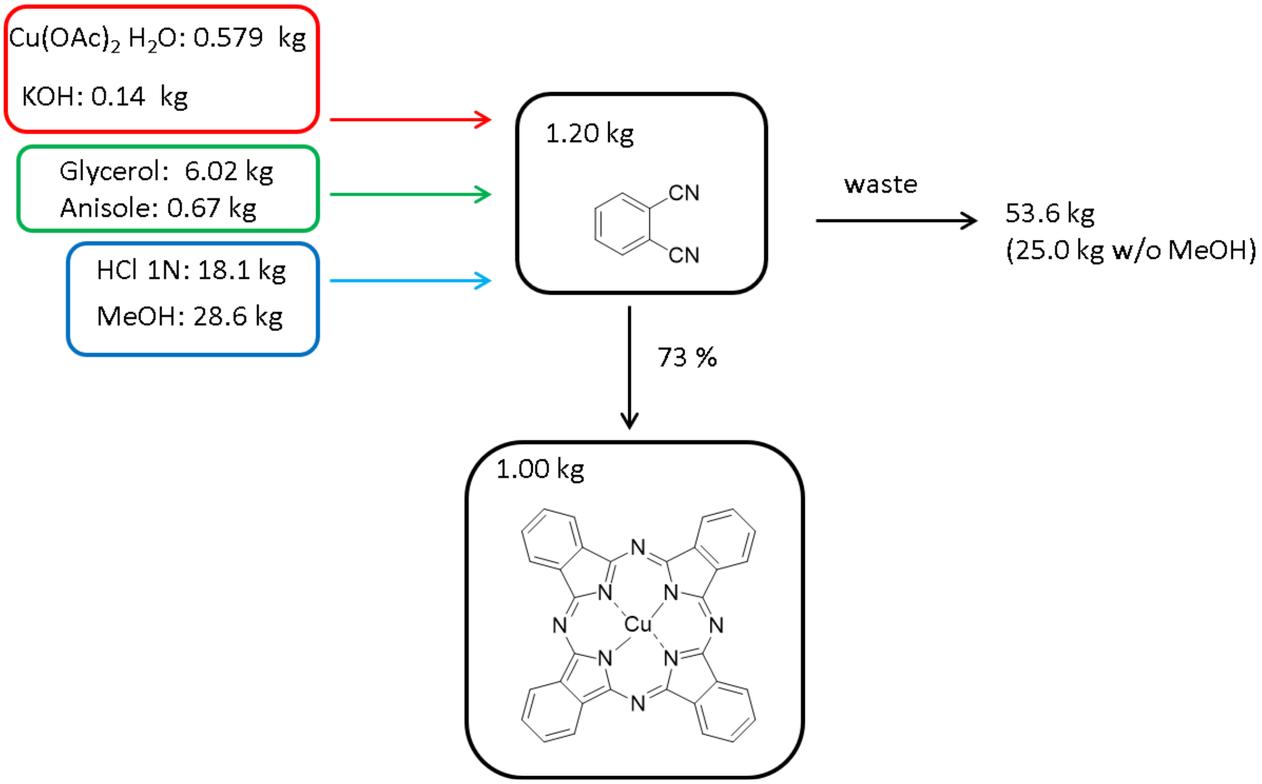

Supplement: Supplementary file 1 [file molecules-26-01760-s001.pdf]
